# Molecular characterization of low-grade serous ovarian carcinoma identifies genomic aberrations according to hormone receptor expression

**DOI:** 10.1038/s41698-022-00288-2

**Published:** 2022-06-29

**Authors:** Dane Cheasley, Marta Llaurado Fernandez, Martin Köbel, Hannah Kim, Amy Dawson, Joshua Hoenisch, Madison Bittner, Derek S. Chiu, Aline Talhouk, C. Blake Gilks, Madawa W. Jayawardana, Kathleen I. Pishas, Anne-Marie Mes-Masson, Diane Provencher, Abhimanyu Nigam, Neville F. Hacker, Kylie L. Gorringe, Ian G. Campbell, Mark S. Carey

**Affiliations:** 1 Cancer Genetics Laboratory, Peter MacCallum Cancer Centre, Melbourne, VIC Australia; 2 Sir Peter MacCallum Department of Oncology, University of Melbourne, Melbourne, VIC Australia; 3 Department of Obstetrics & Gynaecology, Faculty of Medicine, University of British Columbia, Vancouver, BC Canada; 4 Department of Pathology and Laboratory Medicine, University of Calgary, Calgary, AB Canada; 5 Department of Molecular Oncology, BC Cancer Agency, Vancouver, BC Canada; 6 Department of Pathology and Laboratory Medicine, University of British Columbia, Vancouver, BC Canada; 7 Peter MacCallum Cancer Centre, Melbourne, VIC Australia; 8 Centre de recherche du Centre hospitalier de l’Université de Montréal (CRCHUM) and the Department of Medicine, Université de Montréal, Montreal, QC Canada; 9 Centre de recherche du Centre hospitalier de l’Université de Montréal (CRCHUM) and the Division of Gynecologic Oncology, Université de Montréal, Montreal, QC Canada; 10 Prince of Wales Clinical School, University of NSW, Sydney, NSW Australia

**Keywords:** Cancer genomics, Ovarian cancer, Next-generation sequencing

## Abstract

Hormone receptor expression is a characteristic of low-grade serous ovarian carcinoma (LGSOC). Studies investigating estrogen receptor (ER) and progesterone receptor (PR) expression levels suggest its prognostic and predictive significance, although their associations with key molecular aberrations are not well understood. As such, we sought to describe the specific genomic profiles associated with different ER/PR expression patterns and survival outcomes in a cohort of patients with advanced disease. The study comprised fifty-five advanced-staged (III/IV) LGSOCs from the Canadian Ovarian Experimental Unified Resource (COEUR) for which targeted mutation sequencing, copy-number aberration, clinical and follow-up data were available. ER, PR, and p16 expression were assessed by immunohistochemistry. Tumors were divided into low and high ER/PR expression groups based on Allred scoring. Copy number analysis revealed that PR-low tumors (Allred score <2) had a higher fraction of the genome altered by copy number changes compared to PR-high tumors (*p* = 0.001), with cancer genes affected within specific loci linked to altered peptidyl-tyrosine kinase, MAP-kinase, and PI3-kinase signaling. Cox regression analysis showed that ER-high (*p* = 0.02), PR-high (*p* = 0.03), stage III disease (*p* = 0.02), low residual disease burden (*p* = 0.01) and normal p16 expression (*p*<0.001) were all significantly associated with improved overall survival. This study provides evidence that genomic aberrations are linked to ER/PR expression in primary LGSOC.

## Introduction

Low-grade serous ovarian carcinoma (LGSOC) is a rare histotype of epithelial ovarian cancer that is distinguished from other types of epithelial ovarian carcinomas by unique genomic aberrations and high levels of hormone receptor expression^1–4^. LGSOC is often diagnosed in advanced stages and is largely unresponsive to standard ovarian cancer chemotherapeutics^5,6^. The disease is more indolent than high-grade serous ovarian carcinoma with 5-year overall survival (OS) rates of approximately 70%, though OS rates are highly dependent on the volume of residual disease after surgery^6,7^. Unfortunately, the majority (70%) of advanced-stage patients will relapse or progress, upon which retreatment with chemotherapy yields objective response rates below 5%^8–10^.

Estrogen receptor (ER) expression is detected in the majority of LGSOC cases (50–90%) and progesterone receptor (PR) expression is less frequently observed (40–50% of cases)^7,11–14^. Hormone receptor expression appears to have both predictive and prognostic significance in this disease^7,13,15^. Utilizing a binary expression scoring system, LGSOC patients whose tumors were ER+/PR+ had a longer median time to progression than those with ER+/PR- tumors, suggesting that low PR expression appears to be associated with worse outcome independent of ER expression^13^. Strengthening this, in patients with advanced LGSOC, high levels of ERα expression as determined by Allred scoring is significantly associated with better overall survival outcomes, while low levels of PR expression are associated with a more aggressive disease course^15^.

It is clear from the above studies that hormone receptor status defines a specific subtype of LGSOC. However, it is unknown how hormone receptor status interacts with other molecular subtypes such as tumors with MAP-kinase (MAPK) pathway mutations, *USP9X* mutations, *CDKN2A* (which encodes for the tumor suppressor protein, p16) alterations and cases with no specific molecular profiles. As such, our study aims to determine whether (i) genomic alterations in advanced LGSOC are associated with ER/PR expression, and (ii) if these alterations can be targeted and/or influence patient outcomes.

## Results

### Molecular associations in relation to ER/PR expression

The demographic and clinical data summarizing the patient population are shown in Table 1. All 55 COEUR patients in this study had advanced disease (FIGO stage III and IV) in keeping with a cohort of patients with LGSOC who are in need of systemic treatment. Table 2 shows correlates by ER/PR two-tier expression category to the fraction of the genome altered (FGA) and p16 expression status (measured by Fisher’s Exact test). Beyond genomic and hormone receptor expression, p16 expression levels were assessed by immunohistochemistry (IHC) as it is clear from multiple studies including our own^1–4^ that cases with abnormal p16 expression (both loss and overexpression) defines its own molecular subtype of LGSOC and has been linked to shorter survival outcome in the large Ovarian Tumor Tissue Analysis consortium study^16^. Furthermore, as p16 inhibits CDK4/6 and slows the cell cycle, this finding has therapeutic relevance in LGSOC as CDK4/6 inhibitors are currently being evaluated in a clinical trial with LGSOC patients (NCT03673124,^17^). ER-high expression was detected in 61.8% of cases (34/55) while PR-high expression was detected in 66.6% of cases (36/54). When assessing combined ER/PR scores, 42.6% of cases (23/54) demonstrated both high ER and PR expression; 18.5% of cases (10/54) were ER-high and PR-low; 24.1% of cases (13/54) were ER-low and PR-high; and 14.8% of cases (8/54) demonstrated both low ER and PR expression. The median FGA score for the COEUR cohort was 11.5%; therefore, an FGA score >11.5% was selected as FGA-high and an FGA score <11.5% was selected as FGA-low. The FGA score for each LGSOC tumor, including the ER and PR Allred category is shown in supplementary table 1. A greater proportion of FGA-high compared to FGA-low cases were observed in cancers with PR-low expression compared to PR-high expression (13/15 vs 10/31; *p* = 0.001), but not in tumors with ER-low expression to ER-high expression (11/16 vs 12/30; *p* = 0.120). Abnormal p16 expression was similar comparing ER-low to ER-high expressing tumors (3/18 vs 7/33; *p* > 0.999), along with PR-low to PR-high expressing tumors (6/16 vs 4/35; *p* = 0.054).

**Table 1:** Demographic Information on COEUR Study Population.

	No (%)
Mean age at diagnosis (years ± SD)	49.5 ± 13.4
*Age*	
≤40 Years	16 (29)
>40 to ≤50 Years	21 (38)
>50 Years	18 (33)
*Stage*	
3	50 (91)
4	5 (9)
*Residuum*	
None/Micro	6 (11)
Optimal (<1 cm)	20 (36)
Suboptimal (>1 cm)	18 (33)
Missing	11 (20)
*Primary treatment*	
None/unknown	1 (2)
Carboplatin/Taxol	39 (71)
Cisplatinum/Taxol	7 (13)
Single Agent Cis/Carbo-platin	3 (5)
Other	5 (9)
*ER Allred score*	
High (=8)	34 (62)
Low (<8)	21 (38)
*PR Allred score*	
High (≥2)	36 (65)
Low (<2)	18 (33)
Missing	1 (2)
*Fraction genome altered*	
Low (<11.5 %)	23 (42)
High (>11.5 %)	23 (42)
Missing	9 (16)
*P16 staining*	
Wild type	41 (75)
Abnormal loss	6 (11)
Abnormal overexpression (‘block’)	4 (7)
Missing	4 (7)

**Table 2:** Molecular correlates of ER/PR expression.

ER Groups	Low (Allred < 8)	High (Allred = 8)	*p-value*
*P16 IHC result*	No. (%)	No. (%)	
Abnormal	3 (17)	7 (21)	
Normal	15 (83)	26 (79)	*p* > 0.999
Total (51^a^)	18	33	
*Fraction genome altered*			
Low (<11.5%)	5 (31)	18 (60)	
High (>11.5%)	11 (69)	12 (40)	*p* = 0.121
Total (46^b^)	16	30	
*PR groups*	**Low** (Allred < 2)	**High** (Allred > 2)	***p-value***
*P16 IHC result*	No. (%)	No. (%)	
Abnormal	6 (38)	4 (11)	
Normal	10 (62)	31 (89)	*p* = 0.054
Total (51^a^)	16	35	
*Fraction genome Altered*			
Low (<11.5 %)	2 (13)	21 (68)	
High (>11.5 %)	13 (87)	10 (32)	*p* = 0.001
Total (46^b^)	15	31	

Statistical testing was performed using the Fisher’s Exact test comparing the percentage differences of p16 IHC expression and the fraction genome altered status in ER-low to ER-high, and PR-low to PR-high tumors. Percentages are calculated according to the total number within each column.

^a^Missing cases = 4.

^b^Missing cases = 9. No. – number.

### Somatic copy number aberration differences

**Fig. 1:**
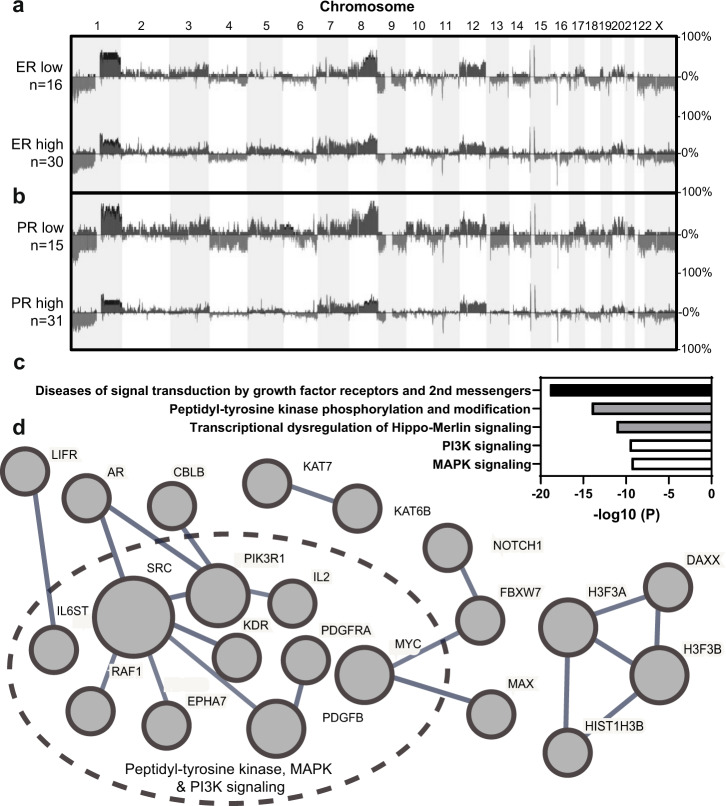
Copy number analysis according to hormone receptor expression status. The frequency of global copy number changes in each chromosome is shown for combined cancer groups comparing (**a**) ER-low to ER-high cancers and (**b**) PR-low to PR-high cancers. CN gains and losses that were statistically significantly different between comparison groups (thresholds of *p* < 0.05 and at least 25% frequency difference) are shown in supplementary table 3. **c** Pathway analysis of all known cancer genes affected within significant CN regions of PR-low compared to PR-high LGSOCs are shown and (**d**) physical protein–protein interactions are shown in network map.

Previously reported genome-wide copy number (CN) data was available for 46 of the LGSOC cases. Comparing ER-low to ER-high LGSOCs showed no significant CN changes between groups (Fig. 1a). Comparing PR-low to PR-high LGSOCs showed widespread significantly different CN losses and gains (Fig. 1b). No significant difference in tumor purity estimates was observed between the comparison groups. CN analysis of each individual ER-low, ER-high, PR-low, and PR-high expressing LGSOC is shown in Supplementary Figs. 1–4. Specific loci differences and known cancer genes affected within regions comparing PR-low to PR-high LGSOCs are shown in Supplementary Table 2.

To determine whether known cancer genes affected within CN regions of PR-low compared to PR-high LGSOCs (*n* = 113/729 cancer genes listed in Cosmic) were enriched within common signaling pathways, pathway enrichment analysis was performed. The genes belonging to each pathway are shown in Supplementary Table 3, and the top 5 cancer signaling pathways and associated Log10(*P*) value are shown in Fig. 1c. The top affected cancer signaling pathways in PR-low cancers included combined diseases of signal transduction by growth factor receptors and second messengers, peptidyl-tyrosine kinase phosphorylation and modification, transcriptional dysregulation of Hippo–Merlin signaling, PI3K signaling and MAPK signaling. From our 113-cancer gene list, we next identified whether any form known physical protein–protein interactions and converted them into a network layout (Fig. 1d). A total of 22/113 proteins form high confidence physical interactions with each other, where 10/22 protein interactions cluster within overlapping peptidyl-tyrosine kinase, MAPK, and the PI3K signaling pathway.

**Fig. 2:**
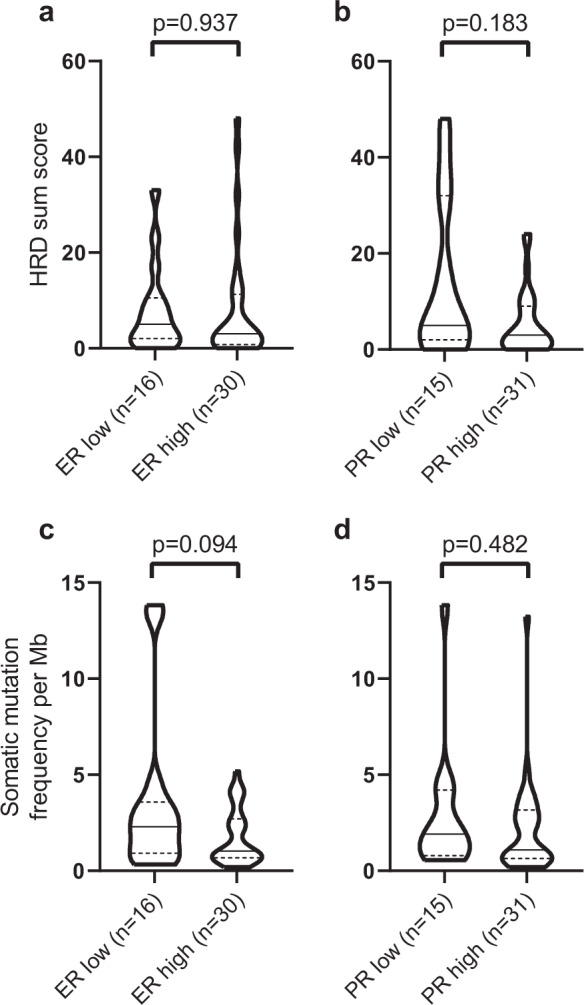
Genomic characteristics defined by hormone receptor expression status. CN profiles were used to generate a homologous recombination deficiency score for each sample and compared between (**a**) ER-low and ER-high cancers and (**b**) PR-low and PR-high cancers. The collective number of coding mutations per/Mb in each sample was compared between (**c**) ER-low and ER-high cancers and (**d**) PR-low and PR-high cancers. Pairwise comparison p-values were calculated using a negative binomial model, adjusted using a false discovery rate method. The black line within the violin plot represents the median. The dashed lines indicate the upper the lower quartiles.

Overall, homologous recombination deficiency (HRD) sum scores were low in the LGSOC cohort with a median HRD score of 3 [range 0–48]. Comparing ER-low to ER-high (Fig. 2a) and PR-low to PR-high (Fig. 2b) LGSOCs showed no significant differences in overall HRD sum score between groups (*p* = 0.937 and *p* = 0.183 respectively; negative binomial regression). Of the six cases with an HRD score of >20, only one case was an ER-high/PR-high (case c556).

### Somatic aberrations according to hormone receptor status

Previously reported targeted exon sequencing of 127 LGSOC driver genes was available for 46 of the COEUR LGSOC cases^1^. We assessed the collective number of all coding mutations per sample as a measure of tumor mutation burden (TMB). The somatic mutation frequency per Mb per tumor was not significantly divergent in ER-low compared with ER-high LGSOCs (Fig. 2c, median 2.30 [range 0.33–13.83] versus median 0.89 [range 0.20–5.19], *p* = 0.094; negative binomial regression). No significant differences were observed comparing PR-low to PR-high LGSOCs (Fig. 2d, median 1.92 [range 0.56–13.83] versus median 0.70 [range 0.20–13.23], *p* = 0.482; negative binomial regression).

The frequency of somatic aberrations in each of the most commonly mutated LGSOC driver genes (*KRAS, BRAF, NRAS, and USP9X)* were similar between ER-low and ER-high cancers with the exception of a higher frequency of *PLEC* mutations (19% versus 0%, *p* = 0.037; two-tailed test), although this difference was not significant after multiple testing correction (*p* = 0.480; Benjamini Hochberg correction) (Table 3). Similar overall frequencies were observed comparing PR-low to PR-high cancers with the exception of *SYNE1* mutations (27% versus 0%, *p* = 0.008; two-tailed test), although similarly this difference was not significant after multiple testing correction (*p* = 0.109; Benjamini Hochberg correction). Whilst both proteins have been linked to epithelial ovarian cancer pathogenesis^18,19^, given that *PLEC* (~4684 amino acids) and *SYNE1* (~8797 amino acids) are large genes and that matching germline DNA was unavailable in this study, we cannot exclude the possibility that the missense variants are germline polymorphisms.

**Table 3:** Somatic driver mutation profile according to low and high hormone receptor groups.

	Estrogen receptor positive								Progesterone receptor positive								Cosmic somatic driver	InTOgen somatic driver	Tumor suppressor gene	Onocgene
	ER-low, *n* = 16			ER-high, *n* = 30					PR-low, *n* = 15			PR-high, *n* = 31								
Gene	Lof	MS	%	Lof	MS	%	*P*	*P* Adj	Lof	MS	%	Lof	MS	%	*P*	*P* Adj				
*KRAS*	–	4	25%	–	9	30%	>0.999	>0.999	–	4	27%	–	9	29%	>0.999	>0.999	Yes	Yes		Yes
*USP9X*	3	1	25%	3	2	17%	0.698	>0.999	–	2	13%	6	1	23%	0.696	>0.999	Yes		Yes	Yes
*NRAS*	–	1	6%	–	4	13%	0.645	>0.999	–	1	7%	–	4	13%	>0.999	>0.999	Yes	Yes		Yes
*BPTF*	–	2	13%	–	2	7%	0.602	>0.999	–	2	13%	–	2	6%	0.587	>0.999				
*BRAF*	–	–	0%	–	4	13%	0.282	0.917	–	1	7%	–	3	10%	>0.999	>0.999	Yes	Yes		Yes
*DNAH10*	–	3	19%	–	1	3%	0.114	0.742	–	–	0%	–	4	13%	0.288	0.748				
*EIF1AX*	–	1	6%	–	3	10%	>0.999	>0.999	–	1	7%	–	3	10%	>0.999	>0.999	Yes	Yes	Yes	
*MACF1*	–	1	6%	–	3	10%	>0.999	>0.999	–	1	7%	–	3	10%	>0.999	>0.999			Yes	Yes
*SYNE1*	–	2	13%	–	2	7%	0.602	>0.999	–	4	27%	–	–	0%	0.008	0.109				
*ARID1A*	2	–	13%	–	1	3%	0.274	0.917	1	1	13%	1	–	3%	0.244	0.748	Yes		Yes	
*CSMD1*	–	–	0%	–	3	10%	0.542	>0.999	–	2	13%	–	1	3%	0.244	0.748			Yes	
*FRAS1*	–	1	6%	–	2	7%	>0.999	>0.999	–	1	7%	–	2	6%	>0.999	>0.999				
*PLEC*	–	3	19%	–	–	0%	0.037	0.48	–	2	13%	–	1	3%	0.244	0.748				

Recurrent somatic mutations are shown. A two-tailed *p*-value (*P*) was calculated comparing the percentage of somatic mutations in ER-low to ER-, and PR-low to PR-high tumors. The *p*-value was adjusted for multiple testing (*P* Adj) using the Benjamini–Hochberg significance test.

*Adj* adjusted, *MS* missense mutation, *LoF* loss-of-function mutation.

### Multivariate Cox regression analysis according to hormone receptor status

Multivariate Cox proportional hazards model was performed to calculate hazard ratios (HR) and their 95% confidence intervals (CI) for the experimental group exposed to the hazard function, with outcome measured for progression-free survival (PFS) and overall survival (OS) (Table 4). The following variables were measured showing the control vs experimental group for age at diagnosis (<40 vs >40 – ≤55; 40 vs >55), stage at diagnosis (III vs IV), disease residuum (none vs <1 cm; none vs >1 cm), ER Allred (low vs high), PR Allred (low vs high) and p16 status (normal vs abnormal). For PFS, stage *(*III vs. IV; HR 3.75 [1.14–12.29], *p =* 0.03), residuum (none vs. >1cm; HR 4.49 [1.18–17.01], *p* < 0.03) and abnormal p16 expression (HR 2.81 [0.98–8.05], *p* = 0.05) were significant independent predictors. For OS, stage (III vs. IV; HR 6.13 [1.29–28.99], *p* = 0.02) residuum (none vs. >1 cm; HR 6.72 [1.61–28.09], *p =* 0.01), ER Allred score (HR 0.32 [0.12–0.8], *p* = 0.02), PR Allred score (HR 0.35 [0.13–0.9], *p =* 0.03), and p16 aberrations (HR 7.78 [2.16–28.05], *p<*0.001) were significant independent variables.

**Table 4:** Multivariable survival analysis (Cox regression) results in stage III/IV LGSOC.

Variable (control vs. experimental)	PFS (median 21 months)		OS (median 72 months)	
	HR (95% CI)	*p-value*	HR (95% CI)	*p-value*
Age at diagnosis (<40 vs >40 - ≤55)	1.08 (0.44-2.63)	0.87	1.45 (0.47-4.48)	0.5
Age at diagnosis (<40 vs >55)	1.33 (0.52-3.4)	0.54	1.8 (0.59-5.52)	0.29
Stage (III vs IV)	3.75 (1.14-12.29)	0.03	6.13 (1.29-28.99)	0.02
Residuum (none vs <1 cm)	0.85 (0.24-3.02)	0.8	1.28 (0.28-5.8)	0.73
Residuum (none vs >1 cm)	4.49 (1.18-17.01)	0.03	6.72 (1.61-28.09)	0.01
ER Allred (low vs high)	0.55 (0.23-1.29)	0.16	0.32 (0.12-0.8)	0.02
PR Allred (low vs high)	0.53 (0.25-1.12)	0.1	0.35 (0.13-0.9)	0.03
p16 (normal vs abnormal)	2.81 (0.98-8.05)	0.05	7.78 (2.16-28.05)	<0.001

*p-values* were calculated using the Wald chi-square test. The median PFS and OS are shown for all 55 advanced-stage cases.

*OS* overall survival, *PFS* progression free survival, *HR* hazard ratio, *CI* confidence interval.

## Discussion

It is clear from this study and others^1–3,15^ that PR-low cases, in particular, define a molecular subtype of LGSOC, confirming an initial observation that ER+/PR- LGSOCs may be associated with a worse clinical outcome compared to ER+/PR+ tumors^13^. Similar to observations in endometrioid ovarian carcinomas^12,20,21^, we found that differences in PR-low compared to PR-high LGSOCs are not driven by differences in somatic mutational changes but display a higher FGA with multiple loci specifically altered. In contrast to endometrioid tumors, in LGSOC the association of genomic CN complexity with PR-low status is not influenced by the presence of *TP53* mutations. Whether individual cancer genes within these regions contribute toward pathogeneses remains unknown. However, based on our in-silico signaling pathway and protein–protein interaction analysis it’s reasonable to speculate that multiple-dosage alterations of genes involved in tyrosine kinase signaling modification may be involved.

PR functions not only as a critical regulator of transcription but also activates signal transduction pathways, many of which are involved in pro-proliferative signaling in ovarian, uterine, and breast cancers^22,23^. Emerging results in breast cancer have identified PR loss as a plausible factor responsible for distinctive tyrosine kinase signaling patterns within HER2-/ER+ breast tumors^24^. Increased kinase activity (and in some cases increased expression) was observed in ER+/HER2−/PR− breast tumors compared to tumors that are positive for PR expression, with perturbations in RAS/MAPK and PI3K signaling being mostly responsible for these differences. Whist the frequency of somatic hotspot mutations in *KRAS, BRAF* and *NRAS* were similar between PR-low and PR-high tumors, the action of PR loss could either be the result from/or be a driver of genomic perturbations in tyrosine kinase signaling, possibly through multiple CN mechanisms particularly involving kinases linked to the MAPK and PI3K pathway. Whilst PR appears to affect high FGA and tyrosine kinase loci, these changes do not appear to be influenced by estrogen-ER activation. However, we also know that ER and PR are intrinsically related to each other, and protein signaling occurs through a network loop. Importantly, ER and PR expression have been implicated in both the maintenance and disruption of genomic integrity through several mechanisms, which include PARP-1 activation^25–27^. This connection makes larger studies looking at ER/PR combinations; the link between hormone receptor expression and CN changes; along with the global assessment of phosphorylated kinase substrates and activated MAPK/PI3K signaling a necessity to understand their importance in LGSOC, particularly in relation to treatment with MEK inhibitor combinations.

Although there is no universally agreed scoring system for ER/PR expression in LGSOC, using the Allred scoring approach we found that cases of advanced-stage LGSOC with higher levels of PR expression by IHC (Allred ≥2) were significantly associated with better OS and PFS, independent of ER expression, disease residuum, and p16 expression. Concordant with our cohort, abnormal p16 expression (both loss and overexpression) has been linked to shorter survival outcome^16^ and appears to define its own molecular subtype independent of hormone receptor status. Furthermore, we found that high ER scoring (Allred ≥8) was also independently associated with better OS. The impact of ER/PR expression on PFS/OS was previously reported using the same group of patients using an ER/PR 3-tiered scoring system^15^. However, this association is apparent using a two-stage scoring system now linked to genomic aberrations in LGSOC.

Due to the rarity of the disease, acquiring sufficient LGSOC samples with matched clinicopathological and survival data is challenging. Given our sample size, the combined findings of this study are useful for hypothesis generation but should be validated in a separate cohort of patients. Additionally, there are limitations to linking genomic changes with phenotype due to the lack of correlation between DNA, RNA, and protein expression. However, it is important to understand how genomic changes correlate with hormone expression to help define molecular subtypes. Perhaps more importantly, our findings will guide future research on how genomic changes impact cellular function in order to define therapeutic vulnerabilities in LGSOC.

In summary, this study shows that advanced-stage patients with PR-low LGSOC are more likely to be FGA high, which may be associated with alterations in tyrosine kinase signaling resulting in a higher likelihood of aggressive disease. Future studies are required to assess the clinical utility of our genomic findings and determine whether low PR is a cause or consequence of genomic changes. This is a pressing unmet research priority given that limited targeted treatment options exists for advanced PR-low tumors and survival rates remain poor for this group.

## Methods

### Low grade serous ovarian cancer cohort

A total of 55 advanced-stage cases (FIGO stage III/IV) from the COEUR^28,29^ were utilized in this study. Clinico-pathological data was collected and survival outcome evaluated (median OS = 72 months, range 1–270 months; median PFS = 21 months, range 1–142 months) for all 55 cases. All tumors were previously characterized as *TP53* wild-type through IHC and somatic mutation profiling, which is a key diagnostic criterion for LGSOC^1,28^. IHC for progesterone receptor expression was assessed across all 55 cases. IHC for estrogen receptor expression was scored on 54/55 cases, as 1 of the LGSOC cases (c2034) was scored from a historical pathology slide and the tumor block was unavailable to section and score PR expression. For somatic mutation and genome-wide CN data, 46/55 LGSOC cases passed QC for Agilent SureSelect library preparation as they had ≥20 ng of tumor DNA, and in a PCR-based quality assay had amplifiable products of ≥200 bp.

The biobanks received ethics approval from their local review boards to collect and share samples and clinical data. All subjects gave broad written consent to future research with their samples and data, without restriction. The study was conducted according to the guidelines of the Declaration of Helsinki. The collection of the COEUR repository samples and data received local ethics approval by the Comité d’éthique de la recherche du CHUM (project reference 39-27-01-2017). Tumor sequencing was approved by the Peter MacCallum Cancer Centre Human Ethics Committee under protocol #09/29. ER and PR IHC and scoring was approved by the institutional human ethics review board at BC Cancer and the University of British Columbia (H14-02859, R05-0119).

### Somatic mutation analysis

Mutation data for 127 recurrently mutated LGSOC genes across all COEUR LGSOC cases in the current study have been reported previously^1^, and includes library construction, sequencing performance and somatic mutation detection.

### Genome-wide copy number analysis

Off-target sequencing reads were used to generate genome-wide CN data for the COEUR cases using CopywriteR^30^ utilizing the female NA12878 control run in the same sequencing batch for normalization^1^. This data was used to study comparisons between defined groups using Nexus^31^, utilizing thresholds of *p* < 0.05 and at least 25% frequency difference, after multiple testing correction.

Genome-wide CN data were also used to calculate a FGA score. FGA was the number of bases affected by CN change for each chromosome divided by the total size into base pairs of that chromosome and then averaged across all chromosomes. The homologous recombination deficiency (HRD) score was calculated for each LGSOC by summing the individual scores for telomeric allelic imbalances, large-scale state transitions, and homologous recombination deficiency-related loss-of-heterozygosity^32^. No significant difference in tumor purity estimates was observed between the comparison groups. Tumor purity for the LGSOC cohort was estimated as follows in order of precedence: (1) If the tumor was driven by a known heterozygous point mutation (*KRAS*, *BRAF,* or *PIK3CA*) and was not CN-amplified at that locus, then that mutation was assumed to be fully clonal and its allele frequency was used as an indicator of purity; (2) manual assessment of CN plots in Nexus; and (3) utilizing the PureCN software package^33^.

### Immunohistochemistry

IHC was performed on whole slide sections from formalin-foxed paraffin-embedded (FFPE) tissues with antibodies frequently used for clinical diagnosis of ovarian cancer^34^. Briefly, 4 μm sections were immuno-stained using DakoCytomation EnVision and System-HRP (Dako Corporation, Carpinteria, CA) or a Leica Bond platform in a two-step technique. Slides were incubated with either anti-ER antibody SP1 (Cat. No. RM-9101, LabVision, Fremont, CA; 1:200 dilution), anti-PR antibody clone E12 (Cat. No. 790-2223, Ventana Medical Systems Inc., Tucson, AZ; 1:200 dilution), anti-p16 (Cat. No. 06695256001, E6H4 clone, CINtec, Roche mtm laboratories; 1:24 dilution) or anti-TP53 (Cat. No. PA0067, clone DO-7, Leica Biosystems, Buffalo Grove, IL; 1:5000 dilution).

### Immunohistochemistry scoring

For ER and PR IHC, whole tumor sections from the COEUR cohort were scored according to the Allred scoring systems^15^. The proportionate score was determined as follows: no tumor cells stained (0 points), <1% (1 point), 1 to 10% (2 points), 10% to 33% (3 points), 33% to 66% (4 points), and >66% (5 points). Intensity scores were categorized as absent (0), weak (1), intermediate (2), and strong staining (3). The proportionate and intensity scores were added to obtain a total score that ranged from 0 to 8. Each tumor was represented by two tissue cores on the COEUR TMA, both were scored and averaged.

For p16 IHC, TMA, and tumor sections were scored and three staining patterns were recorded: abnormal loss, normal expression, and abnormal overexpression (‘block’)^34^. Overexpression was distinguished from heterogeneous staining by using the recommendation for p16 interpretation from The Lower Anogenital Squamous Terminology Standardization Project for HPV-Associated Lesions (LAST Project)^16,35^; that is, overexpression is characterized by diffuse staining of tumor cells in nuclear and/or cytoplasmic compartment with at least moderate intensity in ≥90% of tumor cells. The loss was categorized as the complete absence of staining; normal staining was defined as patchy or mosaic-type in <90% of tumor cells. Each tumor was scored independently by two pathologists and averaged.

### Pathway analysis

Metascape pathway analysis^36^ was performed to determine whether all known cancer genes affected within significant regions of CN loss or gain in PR-low compared to PR-high LGSOCs (*n* = 113/729 genes, identified in Cosmic database^37^) were enriched within common signaling pathways. For each given gene, pathway analysis was carried out within Metascape utilizing the following ontology sources: GO Biological Processes, Reactome Gene Sets, KEGG Pathway, WikiPathways, Canonical Pathways, Hallmark Gene Sets, BioCarta Gene Sets and PANTHER Pathway. Terms with a hypergeometric *p*-value < 0.01, a minimum count within 3 ontology sources, and an enrichment factor >5 (ratio between the observed counts and the counts expected by chance) were collected and grouped into clusters based on their membership similarities. Kappa scores were used as the similarity metric when performing hierarchical clustering on the enriched terms, and sub-trees with a similarity of >0.3 are considered a cluster. For our gene list, protein–protein interaction enrichment analysis was carried out with the STRING^38^ and BioGrid^39^ databases. Only high confidence physical interactions (interaction score >0.9 and 1% false discovery rate) were applied to resultant network maps.

### Statistical analysis

Data were analyzed using R studio (version 1.4.1717) and SPSS statistical software (version 25). As the goal of this study was to evaluate molecular correlates of ER/PR expression, and recognize the limited sample size, we elected to categorize ER and PR into high and low expression categories respectively. This distinction is supported by our previous work^15^ and the work of others^40^ supporting a high threshold for ER IHC expression and a low threshold for PR IHC expression. Thus, those patients with an ER-Allred score of 8 constituted the ER-high group (see Table 1), while those patients with a score of less than 8 categorized as ER-low. For PR, those patients with an Allred score of less than 2 were classified as the PR-low group, while the PR-high group had a score of 2 or greater.

For survival analysis, PFS was calculated from the date of primary surgery to the date of disease progression or death, whichever occurred first. Progression criteria were retrospectively evaluated and based on clinical notes and imaging studies reported by the treating physician. OS was calculated from the date of primary surgery to the date of death, or the last follow-up visit (as a censored observation if the patient was still alive). Multivariate Cox proportional hazards models were used to calculate adjusted HR and their 95% CI for the experimental group exposed to the hazard function. This model was performed accounting for the covariates age, stage, residuum, p16 aberration and ER/PR IHC staining for both PFS and OS. An HR score greater than 1 with a significant *p*-value (<0.05) suggests an increased relative risk associated with the event occurring in the experimental group compared to the control group, and a hazard ratio below 1 with a significant *p*-value suggests a lower risk in the experimental group. The proportionality assumption of the models was checked using the Grambsch–Therneau test^41^ and no violations were found. Variables were included in the multivariable analysis if on univariate testing the *p-*value was less than 0.1. To evaluate the potential impact of missing residuum data on 11 patients, we performed imputation modeling using the MICE algorithm^42^. Statistical testing results were considered significant if the *p*-value was <0.05.

### Reporting summary

Further information on research design is available in the Nature Research Reporting Summary linked to this article.

## Supplementary information

Supplementary Information

REPORTING SUMMARY

## Data Availability

The datasets analyzed during the current study is available from COEUR. Requests to access the entire low-grade serous ovarian cancer cohort or individual samples (using the unique identifiers shown in Supplementary Table 1) is subject to review by the COEUR Study Committee. Following review, there are no restrictions on access to the COEUR datasets such as non-commercial use only or data usage agreement.
