# Supplementary material for: Molecular characterization of low-grade serous ovarian carcinoma identifies genomic aberrations according to hormone receptor expression
Source: NPJ Precis Oncol. 2022 Jun 29;6:47. doi: 10.1038/s41698-022-00288-2 (PMC9242985; doi:10.1038/s41698-022-00288-2)
Supplement: Supplementary file 1 — Supplementary Information [file 41698_2022_288_MOESM1_ESM.pdf]

a

b

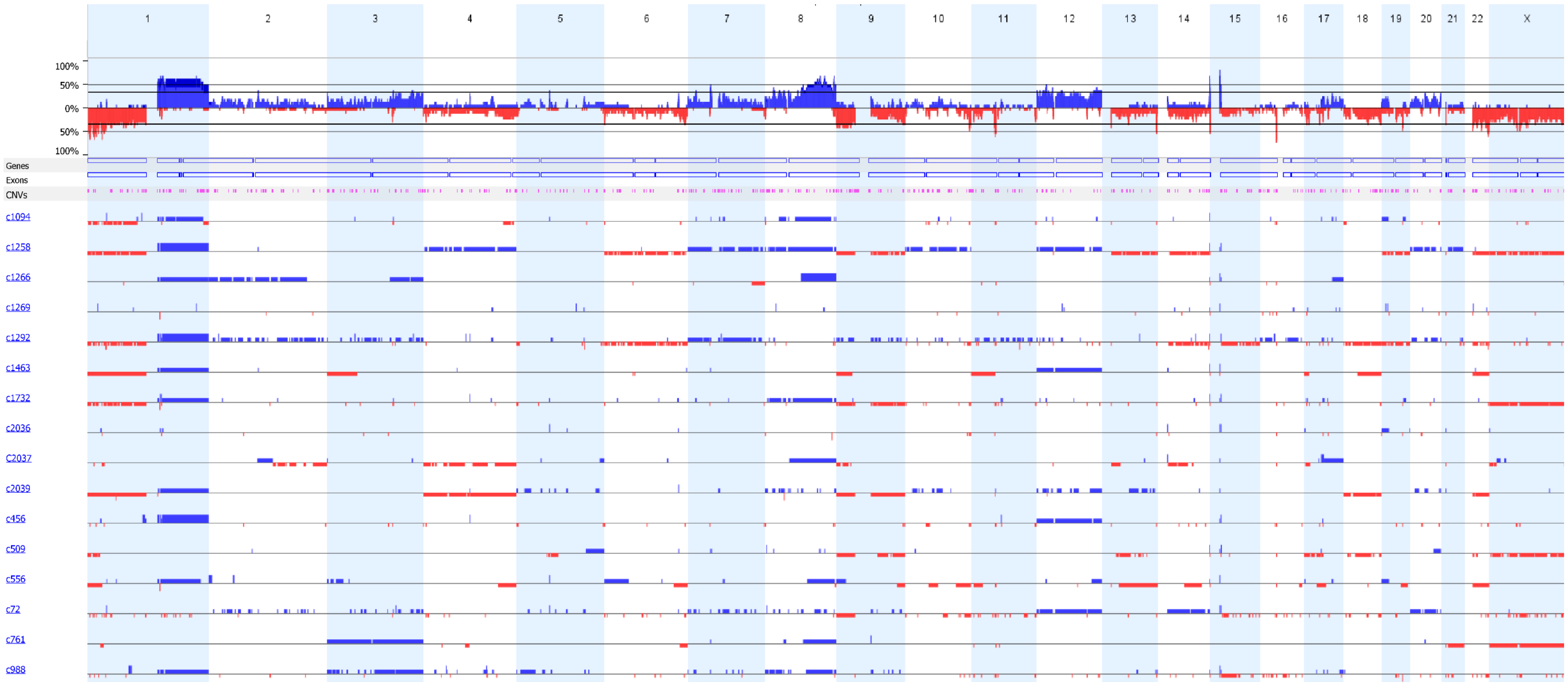

Supplementary figure 1. Copy number analysis of each individual ER-low expressing low-grade serous ovarian carcinoma. (a) The frequency of global copy number changes across each chromosome is shown for combined ER-low cancers (n=16). Copy number gains and losses are shown for each of the 16 ER-low cancers. Individual COEUR sample IDs are shown in the left column. Copy number gain is highlighted in blue, and copy number loss highlighted in red

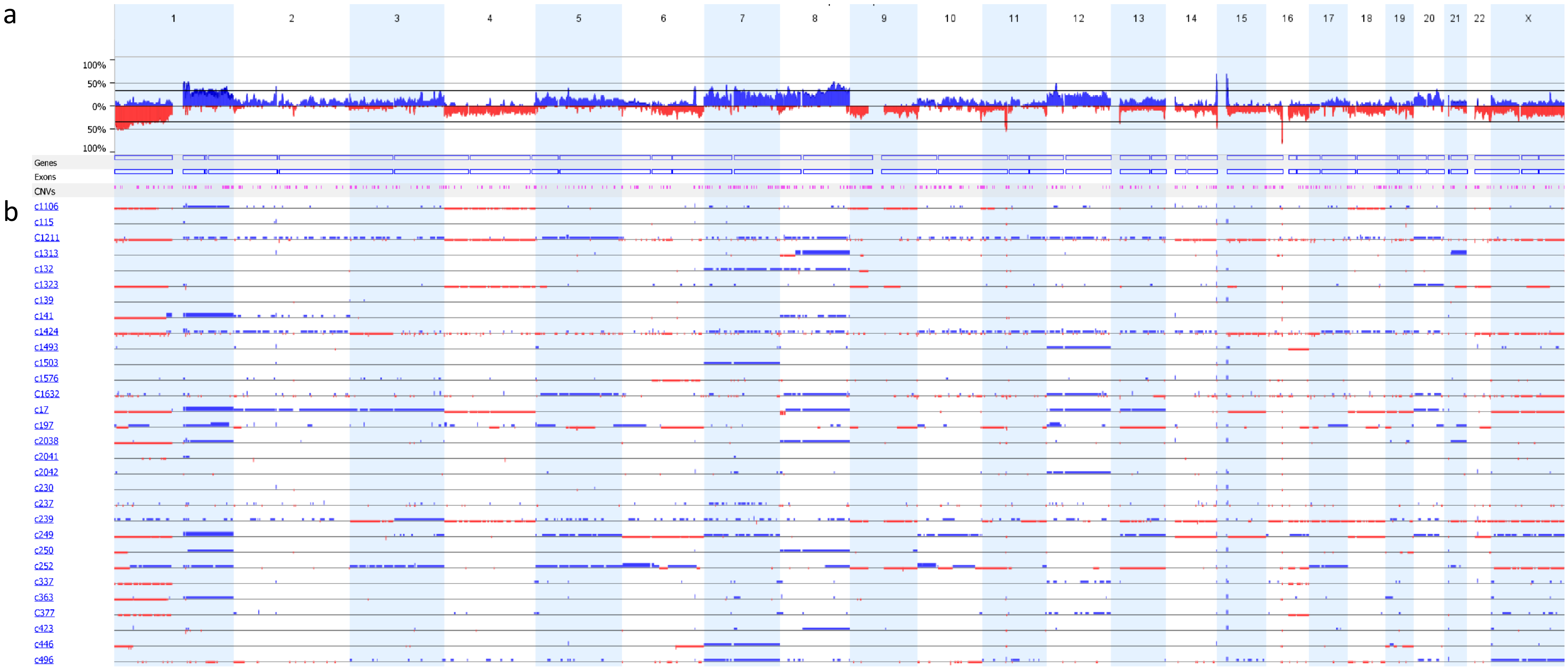

Supplementary figure 2. Copy number analysis of each individual ER-high expressing low-grade serous ovarian carcinoma. (a) The frequency of global copy number changes across each chromosome is shown for combined ER-high cancers (n=30). Copy number gains and losses are shown for each of the 30 ER-high cancers. Individual COEUR sample IDs are shown in the left column. Copy number gain is highlighted in blue, and copy number loss highlighted in red

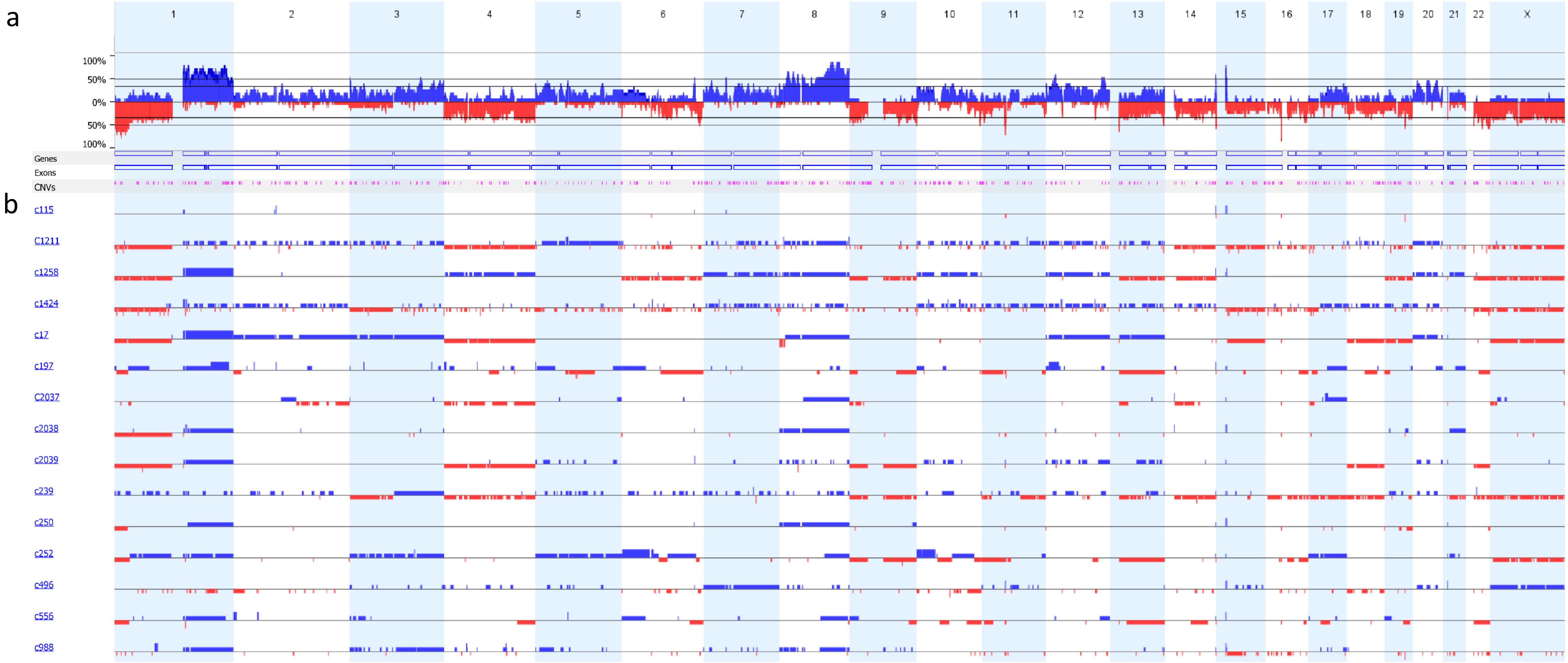

Supplementary figure 3. Copy number analysis of each individual PR-low expressing low-grade serous ovarian carcinoma. (a) The frequency of global copy number changes across each chromosome is shown for combined PR-low cancers (n=15). Copy number gains and losses are shown for each of the 15 PR-low cancers. Individual COEUR sample IDs are shown in the left column. Copy number gain is highlighted in blue, and copy number loss highlighted in red

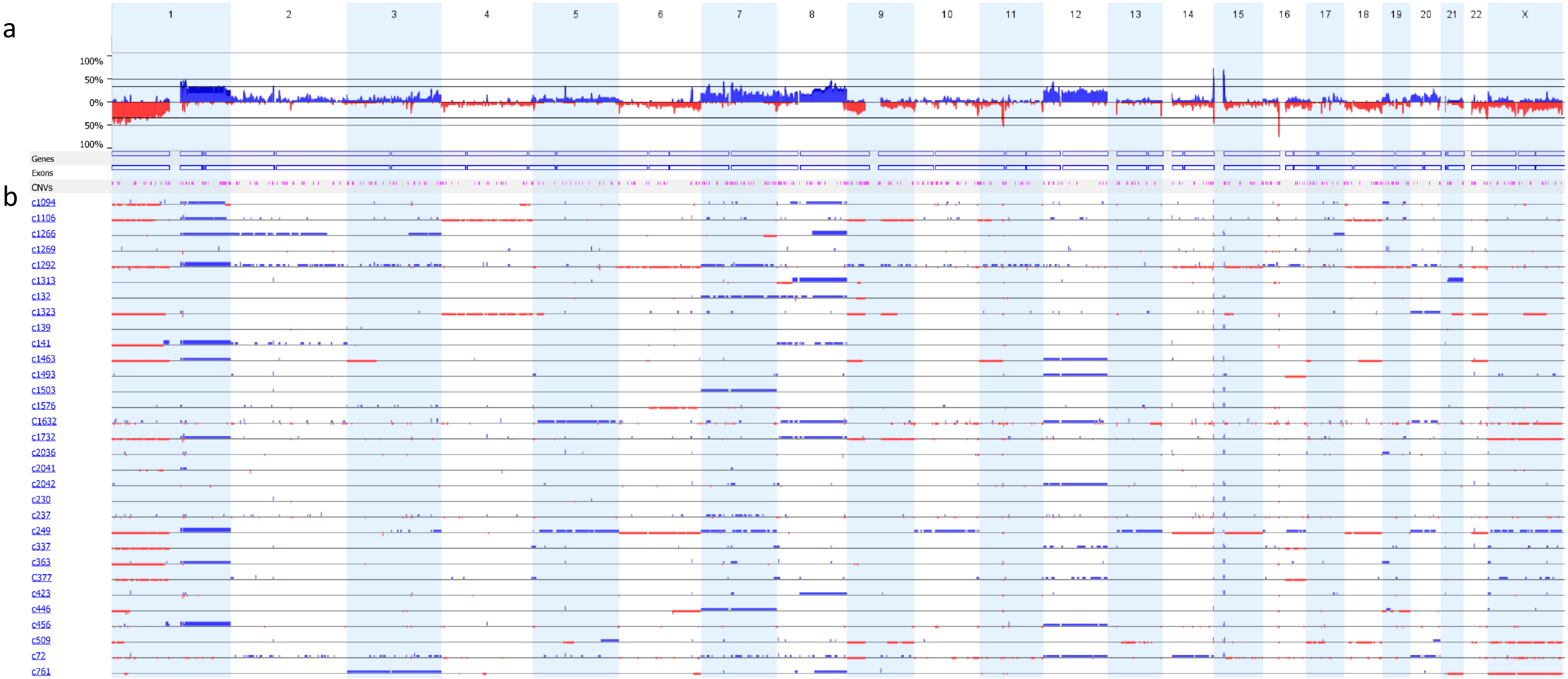

Supplementary figure 4. Copy number analysis of each individual PR-high expressing low-grade serous ovarian carcinoma. (a) The frequency of global copy number changes across each chromosome is shown for combined PR-high cancers (n=31). Copy number gains and losses are shown for each of the 31 PR-high cancers. Individual COEUR sample IDs are shown in the left column. Copy number gain is highlighted in blue, and copy number loss highlighted in red

Supplementary table 1. Fraction of the genome altered score

COEUR sample ID	ER Allred score	PR Allred score	Fraction of the genome altered
c1424	High	Low	54.65%
c1211	High	Low	53.93%
c239	High	Low	52.65%
c1258	Low	Low	51.16%
c17	High	Low	47.72%
c249	High	High	45.54%
c1292	Low	High	43.22%
c252	High	Low	41.99%
c197	High	Low	33.66%
c1632	High	High	31.63%
c2039	Low	Low	27.27%
c72	Low	High	25.72%
c556	Low	Low	23.45%
c496	High	Low	22.12%
c1323	High	High	21.88%
c1106	High	High	19.58%
c1463	Low	High	18.77%
c1732	Low	High	18.76%
c988	Low	Low	18.36%
c509	Low	High	16.52%
c2037	Low	Low	15.99%
c761	Low	High	14.99%
c2038	High	Low	12.78%
c1266	Low	High	10.94%
c1094	Low	High	10.84%
c446	High	High	10.38%
c456	Low	High	9.26%
c250	High	Low	9.09%
c377	High	High	8.91%
c141	High	High	8.23%
c1313	High	High	8.19%
c132	High	High	8.16%
c1493	High	High	7.65%
c337	High	High	7.56%
c237	High	High	7.44%
c363	High	High	6.52%
c2042	High	High	6.10%
c1576	High	High	5.11%
c423	High	High	4.57%
c1503	High	High	4.37%
c2036	Low	High	2.82%
c1269	Low	High	2.45%
c2041	High	High	1.12%
c115	High	Low	0.46%
c139	High	High	0.44%
c230	High	High	0.31%

Supplementary table 2. Comparative loci differences PR low to PR high expression groups.

The frequency of global copy number changes was scored comparing PR-low to PR-high cancers. CN gains and losses (referred to as CN event) that were statistically significantly different between PR-low to PR-high cancers are shown (thresholds of p<0.05 and at least 25% frequency difference). Associated chromosome number, genome location according to the human reference (GRCh38) genome build, cytoband location and length on CN event is shown. The count on the number of genes affected within regions of significant CN differences is shown. Additionally, the count on the number of affected cancer genes and their symbols are shown, which were identified though matching to the Cosmic database of known oncogenes and tumour suppressor genes

Region	Cytoband Location	CN Event	Region Length of CN Event	Freq. in PR Low (%)	Freq. in PR High (%)	Difference	p-value	Count of Gene Symbols	Count of Cosmic Cancer Gene Census Symbols	List of affected cancer genes
chr3:1,550,000-1,700,000	p26.3	CN Gain	150000	53	3	50	0.000	0	0	
chr13:35,375,000-36,550,000	q13.2 - q13.3	CN Loss	1175000	40	0	40	0.001	4	1	NBEA
chr4:156,250,000-157,250,000	q32.1	CN Loss	1000000	40	0	40	0.001	7	0	
chr4:158,300,000-159,300,000	q32.1	CN Loss	1000000	40	0	40	0.001	4	0	
chr4:180,350,000-180,850,000	q34.3	CN Loss	500000	40	0	40	0.001	0	0	
chr13:20,050,000-20,100,000	q12.11	CN Loss	50000	53	6	47	0.001	1	0	
chr3:1,250,000-1,550,000	p26.3	CN Gain	300000	53	6	47	0.001	1	0	
chr3:1,700,000-2,050,000	p26.3	CN Gain	350000	47	3	43	0.001	0	0	
chr4:161,600,000-162,800,000	q32.1 - q32.2	CN Loss	1200000	47	3	43	0.001	1	0	
chr8:119,750,000-120,750,000	q24.12	CN Gain	1000000	87	32	54	0.001	9	0	
chr8:123,200,000-126,750,000	q24.13	CN Gain	3550000	87	32	54	0.001	40	0	
chr8:107,150,000-108,450,000	q23.1	CN Gain	1300000	87	35	51	0.001	3	0	
chr8:110,400,000-111,150,000	q23.1 - q23.2	CN Gain	750000	87	35	51	0.001	5	0	
chr8:16,150,000-16,600,000	p22	CN Gain	450000	60	13	47	0.002	0	0	
chr8:20,200,000-20,250,000	p21.3	CN Gain	50000	60	13	47	0.002	0	0	
chr10:15,450,000-15,500,000	p13	CN Gain	50000	33	0	33	0.002	0	0	
chr10:7,850,000-7,950,000	p14	CN Gain	100000	33	0	33	0.002	1	0	
chr12:104,050,000-104,100,000	q23.3	CN Loss	50000	33	0	33	0.002	1	0	
chr13:23,100,000-28,950,000	q12.11 - q12.3	CN Loss	5850000	33	0	33	0.002	58	2	CDX2, FLT3
chr13:59,150,000-64,900,000	q21.1 - q21.31	CN Loss	5750000	33	0	33	0.002	20	0	
chr13:69,650,000-70,300,000	q21.33	CN Loss	650000	33	0	33	0.002	2	0	
chr13:82,300,000-83,700,000	q31.1	CN Loss	1400000	33	0	33	0.002	0	0	
chr17:12,700,000-12,850,000	p12	CN Loss	150000	33	0	33	0.002	2	0	
chr4:117,350,000-119,150,000	q26	CN Loss	1800000	33	0	33	0.002	6	0	
chr4:120,450,000-121,050,000	q26 - q27	CN Loss	600000	33	0	33	0.002	4	0	
chr4:157,250,000-158,300,000	q32.1	CN Loss	1050000	33	0	33	0.002	3	0	
chr6:24,250,000-25,950,000	p22.3 - p22.2	CN Gain	1700000	33	0	33	0.002	23	0	
chr13:20,000,000-20,050,000	q12.11	CN Loss	50000	53	10	44	0.002	1	0	
chr3:950,000-1,250,000	p26.3	CN Gain	300000	53	10	44	0.002	1	0	
chr8:143,150,000-143,450,000	q24.3	CN Gain	300000	67	19	47	0.003	3	0	
chr8:20,100,000-20,150,000	p21.3	CN Gain	50000	67	19	47	0.003	2	0	
chr8:28,750,000-31,050,000	p21.1 - p12	CN Gain	2300000	67	19	47	0.003	22	2	LEPROTL1, WRN
chr11:6,200,000-6,250,000	p15.4	CN Loss	50000	47	6	40	0.003	3	0	
chr13:20,100,000-20,150,000	q12.11	CN Loss	50000	47	6	40	0.003	1	0	
chr4:104,100,000-104,750,000	q24	CN Loss	650000	47	6	40	0.003	3	0	
chr4:160,300,000-161,600,000	q32.1	CN Loss	1300000	47	6	40	0.003	1	0	
chr4:163,325,000-164,000,000	q32.2	CN Loss	675000	47	6	40	0.003	0	0	
chr4:172,850,000-175,150,000	q34.1	CN Loss	2300000	47	6	40	0.003	12	0	
chr4:179,850,000-180,250,000	q34.3	CN Loss	400000	47	6	40	0.003	0	0	
chr4:189,200,000-191,154,276	q35.2	CN Loss	1954276	47	6	40	0.003	9	1	DUX4L1
chr4:44,450,000-45,200,000	p13 - p12	CN Loss	750000	47	6	40	0.003	4	0	
chr13:29,400,000-30,050,000	q12.3	CN Loss	650000	40	3	37	0.003	1	0	
chr13:31,800,000-32,500,000	q12.3 - q13.1	CN Loss	700000	40	3	37	0.003	3	0	
chr13:34,950,000-35,375,000	q13.2	CN Loss	425000	40	3	37	0.003	2	0	
chr13:36,550,000-38,650,000	q13.3	CN Loss	2100000	40	3	37	0.003	19	0	
chr13:55,450,000-55,500,000	q21.1	CN Loss	50000	40	3	37	0.003	0	0	
chr3:125,450,000-125,500,000	q21.2	CN Gain	50000	40	3	37	0.003	1	0	
chr3:2,050,000-2,250,000	p26.3	CN Gain	200000	40	3	37	0.003	2	0	
chr4:103,150,000-103,950,000	q24	CN Loss	800000	40	3	37	0.003	10	0	
chr4:113,450,000-114,900,000	q25 - q26	CN Loss	1450000	40	3	37	0.003	14	0	
chr4:123,250,000-123,450,000	q27	CN Loss	200000	40	3	37	0.003	3	1	IL2
chr4:156,050,000-156,250,000	q32.1	CN Loss	200000	40	3	37	0.003	1	0	
chr4:159,300,000-159,750,000	q32.1	CN Loss	450000	40	3	37	0.003	5	0	
chr4:162,800,000-163,100,000	q32.2	CN Loss	300000	40	3	37	0.003	2	0	
chr4:180,250,000-180,350,000	q34.3	CN Loss	100000	40	3	37	0.003	0	0	
chr4:180,850,000-181,750,000	q34.3	CN Loss	900000	40	3	37	0.003	0	0	
chr4:55,850,000-56,150,000	q12	CN Loss	300000	40	3	37	0.003	1	1	KDR
chr9:107,350,000-107,450,000	q31.1	CN Loss	100000	40	3	37	0.003	3	0	
chr8:142,400,000-142,450,000	q24.3	CN Gain	50000	73	26	48	0.004	2	0	
chr8:99,950,000-100,650,000	q22.2	CN Gain	700000	73	26	48	0.004	5	0	
chr8:111,150,000-111,450,000	q23.2	CN Gain	300000	87	39	48	0.004	0	0	
chr8:111,900,000-111,950,000	q23.2	CN Gain	50000	87	39	48	0.004	1	0	
chr8:120,750,000-122,200,000	q24.12	CN Gain	1450000	87	39	48	0.004	8	0	
chr8:122,600,000-123,200,000	q24.13	CN Gain	600000	87	39	48	0.004	2	0	
chr1:185,100,000-186,100,000	q25.3 - q31.1	CN Gain	1000000	80	32	48	0.004	6	0	
chr1:228,750,000-233,050,000	q42.13 - q42.2	CN Gain	4300000	80	32	48	0.004	173	0	
chr8:105,850,000-107,150,000	q22.3 - q23.1	CN Gain	1300000	80	32	48	0.004	2	0	
chr8:119,050,000-119,750,000	q24.11 - q24.12	CN Gain	700000	80	32	48	0.004	3	1	EXT1
chr8:112,450,000-112,500,000	q23.3	CN Gain	50000	87	42	45	0.005	1	0	
chr8:122,200,000-122,600,000	q24.12 - q24.13	CN Gain	400000	87	42	45	0.005	0	0	
chr8:16,600,000-16,700,000	p22	CN Gain	100000	60	16	44	0.005	0	0	
chr8:28,050,000-28,150,000	p21.1	CN Gain	100000	60	16	44	0.005	0	0	
chr8:39,350,000-39,400,000	p11.22	CN Gain	50000	60	16	44	0.005	1	0	
chr8:16,700,000-16,750,000	p22	CN Gain	50000	67	23	44	0.008	0	0	
chr8:18,850,000-20,100,000	p22 - p21.3	CN Gain	1250000	67	23	44	0.008	8	0	
chr11:6,100,000-6,200,000	p15.4	CN Loss	100000	47	10	37	0.008	2	0	
chr22:43,400,000-43,600,000	q13.2	CN Loss	200000	47	10	37	0.008	8	0	
chr4:164,000,000-164,200,000	q32.2	CN Loss	200000	47	10	37	0.008	2	0	
chr4:171,000,000-172,850,000	q33 - q34.1	CN Loss	1850000	47	10	37	0.008	8	0	
chr4:178,300,000-179,850,000	q34.3	CN Loss	1550000	47	10	37	0.008	3	0	
chr8:24,250,000-24,350,000	p21.2	CN Gain	100000	47	10	37	0.008	4	0	
chr8:33,500,000-34,000,000	p12	CN Gain	500000	47	10	37	0.008	0	0	
chr9:105,850,000-106,700,000	q31.1	CN Loss	850000	47	10	37	0.008	1	0	
chr9:139,100,000-139,250,000	q34.3	CN Loss	150000	47	10	37	0.008	3	0	
chrX:139,800,000-139,950,000	q27.1	CN Loss	150000	47	10	37	0.008	2	0	
chrX:7,350,000-7,650,000	p22.31	CN Loss	300000	47	10	37	0.008	0	0	
chr1:60,000,000-60,550,000	p32.1	CN Gain	550000	27	0	27	0.008	6	0	
chr10:11,050,000-13,150,000	p14 - p13	CN Gain	2100000	27	0	27	0.008	20	0	
chr10:15,500,000-15,550,000	p13	CN Gain	50000	27	0	27	0.008	0	0	
chr10:18,700,000-19,300,000	p12.31	CN Gain	600000	27	0	27	0.008	3	0	
chr10:7,950,000-8,750,000	p14	CN Gain	800000	27	0	27	0.008	5	1	GATA3
chr11:67,800,000-68,750,000	q13.2 - q13.3	CN Gain	950000	27	0	27	0.008	16	0	
chr12:104,100,000-104,150,000	q23.3	CN Loss	50000	27	0	27	0.008	1	0	
chr13:114,500,000-115,169,878	q34	CN Loss	669878	27	0	27	0.008	14	0	
chr13:64,900,000-67,500,000	q21.31 - q21.32	CN Loss	2600000	27	0	27	0.008	5	0	

chr13:70,300,000-74,800,000	q21.33 - q22.1	CN Loss	4500000	27	0	27	0.008	11	0	
chr13:85,950,000-89,150,000	q31.1 - q31.2	CN Loss	3200000	27	0	27	0.008	8	0	
chr16:18,150,000-18,475,000	p12.3	CN Loss	325000	27	0	27	0.008	12	0	
chr16:7,150,000-7,650,000	p13.3	CN Loss	500000	27	0	27	0.008	1	0	
chr17:12,850,000-13,200,000	p12	CN Loss	350000	27	0	27	0.008	2	0	
chr17:42,300,000-45,550,000	q21.31 - q21.32	CN Gain	3250000	27	0	27	0.008	77	0	
chr17:49,450,000-49,550,000	q21.33	CN Gain	100000	27	0	27	0.008	1	0	
chr17:54,650,000-56,650,000	q22	CN Gain	2000000	27	0	27	0.008	38	2	MSI2, RNF43
chr2:50,350,000-50,400,000	p16.3	CN Gain	50000	27	0	27	0.008	1	0	
chr3:76,250,000-76,550,000	p12.3	CN Loss	300000	27	0	27	0.008	0	1	ROBO2
chr4:119,150,000-120,450,000	q26	CN Loss	1300000	27	0	27	0.008	19	0	
chr4:125,200,000-125,500,000	q28.1	CN Gain	300000	27	0	27	0.008	1	0	
chr4:143,850,000-144,700,000	q31.21	CN Gain	850000	27	0	27	0.008	8	0	
chr4:94,800,000-95,050,000	q22.2	CN Loss	250000	27	0	27	0.008	1	0	
chr6:13,000,000-24,250,000	p24.1 - p22.3	CN Gain	11250000	27	0	27	0.008	51	1	DEK
chr6:31,425,000-35,750,000	p21.33 - p21.31	CN Gain	4325000	27	0	27	0.008	190	3	DAXX, HMGA1, FANCE
chr6:750,000-800,000	p25.3	CN Gain	50000	27	0	27	0.008	0	0	
chr6:76,000,000-76,650,000	q14.1	CN Gain	650000	27	0	27	0.008	7	0	
chr22:35,400,000-35,500,000	q12.3	CN Loss	100000	60	19	41	0.009	1	1	ISX
chr8:143,050,000-143,150,000	q24.3	CN Gain	100000	60	19	41	0.009	1	0	
chr8:143,450,000-143,550,000	q24.3	CN Gain	100000	60	19	41	0.009	2	0	
chr8:20,150,000-20,200,000	p21.3	CN Gain	50000	60	19	41	0.009	1	0	
chr8:28,150,000-28,750,000	p21.1	CN Gain	600000	60	19	41	0.009	9	0	
chr8:37,450,000-37,600,000	p11.23	CN Gain	150000	60	19	41	0.009	4	0	
chr8:14,750,000-16,150,000	p22	CN Gain	1400000	53	13	40	0.009	3	0	
chr8:141,100,000-141,400,000	q24.3	CN Gain	300000	53	13	40	0.009	2	0	
chr8:20,250,000-20,300,000	p21.3	CN Gain	50000	53	13	40	0.009	0	0	
chr8:27,400,000-27,500,000	p21.1	CN Gain	100000	53	13	40	0.009	4	0	
chr9:103,750,000-104,400,000	q31.1	CN Loss	650000	53	13	40	0.009	10	0	
chr10:59,850,000-60,550,000	q21.1	CN Gain	700000	40	6	34	0.010	7	0	
chr10:73,550,000-75,600,000	q22.1 - q22.2	CN Gain	2050000	40	6	34	0.010	41	0	
chr11:6,250,000-7,550,000	p15.4	CN Loss	1300000	40	6	34	0.010	35	0	
chr12:103,450,000-103,500,000	q23.2	CN Loss	50000	40	6	34	0.010	0	0	
chr13:20,150,000-20,200,000	q12.11	CN Loss	50000	40	6	34	0.010	1	0	
chr13:55,400,000-55,450,000	q21.1	CN Loss	50000	40	6	34	0.010	0	0	
chr14:25,100,000-26,050,000	q12	CN Loss	950000	40	6	34	0.010	2	0	
chr14:66,200,000-66,850,000	q23.3	CN Loss	650000	40	6	34	0.010	1	0	
chr14:71,950,000-73,350,000	q24.2	CN Loss	1400000	40	6	34	0.010	5	0	
chr14:79,400,000-79,700,000	q31.1	CN Loss	300000	40	6	34	0.010	2	0	
chr16:73,500,000-74,200,000	q22.3 - q23.1	CN Loss	700000	40	6	34	0.010	0	0	
chr16:81,925,000-82,200,000	q23.3	CN Loss	275000	40	6	34	0.010	4	0	
chr17:73,950,000-74,800,000	q25.1 - q25.2	CN Gain	850000	40	6	34	0.010	37	1	SRSF2
chr19:56,200,000-56,300,000	q13.42 - q13.43	CN Loss	100000	40	6	34	0.010	5	0	
chr3:124,400,000-124,450,000	q21.2	CN Gain	50000	40	6	34	0.010	2	0	
chr4:100,950,000-101,600,000	q23 - q24	CN Loss	650000	40	6	34	0.010	6	0	
chr4:103,950,000-104,100,000	q24	CN Loss	150000	40	6	34	0.010	3	0	
chr4:104,750,000-109,350,000	q24 - q25	CN Loss	4600000	40	6	34	0.010	24	2	TET2, LEF1
chr4:110,650,000-113,450,000	q25	CN Loss	2800000	40	6	34	0.010	17	0	
chr4:114,900,000-115,050,000	q26	CN Loss	150000	40	6	34	0.010	1	0	
chr4:122,250,000-123,250,000	q27	CN Loss	1000000	40	6	34	0.010	9	0	
chr4:130,350,000-130,900,000	q28.2	CN Loss	550000	40	6	34	0.010	2	0	
chr4:131,200,000-132,450,000	q28.3	CN Loss	1250000	40	6	34	0.010	1	0	
chr4:14,450,000-14,800,000	p15.33	CN Loss	350000	40	6	34	0.010	1	0	
chr4:147,250,000-147,350,000	q31.22	CN Loss	100000	40	6	34	0.010	2	0	
chr4:149,500,000-151,900,000	q31.23 - q31.3	CN Loss	2400000	40	6	34	0.010	7	0	
chr4:153,150,000-156,050,000	q31.3 - q32.1	CN Loss	2900000	40	6	34	0.010	25	1	FBXW7
chr4:159,750,000-160,300,000	q32.1	CN Loss	550000	40	6	34	0.010	5	0	
chr4:163,100,000-163,325,000	q32.2	CN Loss	225000	40	6	34	0.010	0	0	
chr4:175,150,000-178,300,000	q34.1 - q34.3	CN Loss	3150000	40	6	34	0.010	16	0	
chr4:181,750,000-182,300,000	q34.3	CN Loss	550000	40	6	34	0.010	2	0	
chr4:186,050,000-189,200,000	q35.1 - q35.2	CN Loss	3150000	40	6	34	0.010	27	1	FAT1
chr4:36,400,000-36,550,000	p14	CN Loss	150000	40	6	34	0.010	2	0	
chr4:43,800,000-44,450,000	p13	CN Loss	650000	40	6	34	0.010	2	0	
chr4:45,200,000-48,100,000	p12	CN Loss	2900000	40	6	34	0.010	15	0	
chr4:54,750,000-55,850,000	q12	CN Loss	1100000	40	6	34	0.010	7	3	CHIC2, PDGFRA, KIT
chr4:67,150,000-67,300,000	q13.2	CN Loss	150000	40	6	34	0.010	0	0	
chr4:75,950,000-76,400,000	q13.3 - q21.1	CN Loss	450000	40	6	34	0.010	3	0	
chr4:79,850,000-83,200,000	q21.21 - q21.22	CN Loss	3350000	40	6	34	0.010	14	0	
chr5:19,900,000-23,350,000	p14.3 - p14.2	CN Gain	3450000	40	6	34	0.010	9	0	
chr5:27,500,000-30,550,000	p14.1 - p13.3	CN Gain	3050000	40	6	34	0.010	6	0	
chr5:53,100,000-53,350,000	q11.2	CN Gain	250000	40	6	34	0.010	3	0	
chr8:34,800,000-35,000,000	p12	CN Gain	200000	40	6	34	0.010	0	0	
chr8:36,650,000-36,700,000	p11.23	CN Gain	50000	40	6	34	0.010	1	0	
chr9:106,800,000-107,350,000	q31.1	CN Loss	550000	40	6	34	0.010	7	0	
chr9:108,400,000-109,350,000	q31.2	CN Loss	950000	40	6	34	0.010	3	1	TAL2
chrX:31,050,000-32,050,000	p21.2 - p21.1	CN Loss	1000000	40	6	34	0.010	2	0	
chrX:4,850,000-5,400,000	p22.32	CN Loss	550000	40	6	34	0.010	0	0	
chrX:5,800,000-6,000,000	p22.32 - p22.31	CN Loss	200000	40	6	34	0.010	1	0	
chr1:181,100,000-181,250,000	q25.3	CN Gain	150000	73	29	44	0.010	2	0	
chr1:182,050,000-182,100,000	q25.3	CN Gain	50000	73	29	44	0.010	0	0	
chr8:100,650,000-101,250,000	q22.2	CN Gain	600000	73	29	44	0.010	7	1	COX6C
chr8:118,750,000-119,000,000	q24.11	CN Gain	250000	73	29	44	0.010	2	1	EXT1
chr8:126,750,000-132,350,000	q24.13 - q24.22	CN Gain	5600000	73	29	44	0.010	33	1	MYC
chr8:134,600,000-135,000,000	q24.22	CN Gain	400000	73	29	44	0.010	3	0	
chr8:145,000,000-146,364,022	q24.3	CN Gain	1364022	73	29	44	0.010	66	1	RECQL4
chr8:111,950,000-112,450,000	q23.2 - q23.3	CN Gain	500000	87	45	42	0.010	3	0	
chr10:0-1,150,000	p15.3	CN Gain	1150000	33	3	30	0.010	13	1	LARP4B
chr10:14,850,000-15,450,000	p13	CN Gain	600000	33	3	30	0.010	14	0	
chr10:25,200,000-25,300,000	p12.1	CN Gain	100000	33	3	30	0.010	2	0	
chr10:28,300,000-29,550,000	p12.1	CN Gain	1250000	33	3	30	0.010	10	0	
chr10:3,950,000-7,850,000	p15.1 - p14	CN Gain	3900000	33	3	30	0.010	47	0	
chr10:34,000,000-35,800,000	p11.22 - p11.21	CN Gain	1800000	33	3	30	0.010	7	0	
chr10:54,450,000-54,950,000	q21.1	CN Gain	500000	33	3	30	0.010	2	0	
chr10:55,150,000-57,650,000	q21.1	CN Gain	2500000	33	3	30	0.010	4	0	
chr10:58,850,000-59,800,000	q21.1	CN Gain	950000	33	3	30	0.010	1	0	
chr10:71,300,000-73,350,000	q22.1	CN Gain	2050000	33	3	30	0.010	24	1	PRF1
chr10:77,450,000-77,850,000	q22.2 - q22.3	CN Gain	400000	33	3	30	0.010	2	0	
chr13:107,600,000-109,400,000	q33.3	CN Loss	1800000	33	3	30	0.010	8	0	
chr13:113,300,000-115,169,878	q34	CN Gain	1869878	33	3	30	0.010	37	0	
chr13:20,250,000-23,100,000	q12.11	CN Loss	2850000	33	3	30	0.010	28	2	ZMYM2, LATS2
chr13:28,950,000-29,400,000	q12.3	CN Loss	450000	33	3	30	0.010	4	0	
chr13:30,050,000-31,800,000	q12.3	CN Loss	1750000	33	3	30	0.010	24	0	
chr13:32,500,000-34,950,000	q13.1 - q13.2	CN Loss	2450000	33	3	30	0.010	17	1	BRCA2
chr13:38,650,000-40,300,000	q13.3 - q14.11	CN Loss	1650000	33	3	30	0.010	12	2	LHFPL6, LHFP
chr13:42,600,000-44,400,000	q14.11	CN Loss	1800000	33	3	30	0.010	12	0	
chr13:45,150,000-48,450,000	q14.11 - q14.2	CN Loss	3300000	33	3	30	0.010	33	1	LCP1
chr13:50,150,000-53,450,000	q14.2 - q14.3	CN Loss	3300000	33	3	30	0.010	48	0	
chr13:55,500,000-55,750,000	q21.1	CN Loss	250000	33	3	30	0.010	1	0	
chr13:57,700,000-59,150,000	q21.1	CN Loss	1450000	33	3	30	0.010	28	0	

chr13:67,500,000-68,450,000	q21.32	CN Loss	950000	33	3	30	0.010	4	0	
chr13:69,300,000-69,650,000	q21.33	CN Loss	350000	33	3	30	0.010	1	0	
chr13:71,100,000-72,800,000	q21.33	CN Gain	1700000	33	3	30	0.010	2	0	
chr13:75,350,000-75,700,000	q22.1 - q22.2	CN Loss	350000	33	3	30	0.010	0	0	
chr13:78,950,000-79,400,000	q22.3 - q31.1	CN Loss	450000	33	3	30	0.010	5	0	
chr13:81,800,000-82,300,000	q31.1	CN Gain	500000	33	3	30	0.010	1	0	
chr13:83,700,000-84,900,000	q31.1	CN Loss	1200000	33	3	30	0.010	2	0	
chr13:87,950,000-89,150,000	q31.2	CN Gain	1200000	33	3	30	0.010	5	0	
chr15:59,150,000-60,000,000	q22.1 - q22.2	CN Loss	850000	33	3	30	0.010	11	0	
chr17:11,800,000-12,700,000	p12	CN Loss	900000	33	3	30	0.010	9	1	MAP2K4
chr17:41,650,000-42,000,000	q21.31	CN Gain	350000	33	3	30	0.010	8	0	
chr17:47,350,000-47,850,000	q21.32 - q21.33	CN Gain	500000	33	3	30	0.010	12	1	SPOP
chr17:49,350,000-49,450,000	q21.33	CN Gain	100000	33	3	30	0.010	3	0	
chr17:56,950,000-57,050,000	q22	CN Gain	100000	33	3	30	0.010	1	0	
chr17:75,450,000-77,050,000	q25.3	CN Gain	1600000	33	3	30	0.010	31	2	SEPT9, CANT1
chr17:77,575,000-81,195,210	q25.3	CN Gain	3620210	33	3	30	0.010	108	2	RNF213, ASPSCR1
chr19:35,000,000-35,400,000	q13.11	CN Loss	400000	33	3	30	0.010	11	0	
chr2:163,900,000-164,100,000	q24.3	CN Gain	200000	33	3	30	0.010	0	0	
chr3:101,800,000-105,250,000	q12.3 - q13.11	CN Gain	3450000	33	3	30	0.010	4	0	
chr3:11,150,000-13,450,000	p25.3 - p25.1	CN Gain	2300000	33	3	30	0.010	18	2	PPARG, RAF1
chr3:122,000,000-123,950,000	q21.1 - q21.2	CN Gain	1950000	33	3	30	0.010	27	0	
chr3:125,500,000-125,600,000	q21.2	CN Gain	100000	33	3	30	0.010	3	0	
chr3:2,250,000-3,350,000	p26.3 - p26.2	CN Gain	1100000	33	3	30	0.010	5	0	
chr3:21,200,000-23,300,000	p24.3	CN Gain	2100000	33	3	30	0.010	6	0	
chr3:24,950,000-25,850,000	p24.2	CN Gain	900000	33	3	30	0.010	6	0	
chr3:8,450,000-9,800,000	p26.1 - p25.3	CN Gain	1350000	33	3	30	0.010	20	1	SRGAP3
chr4:101,850,000-103,150,000	q24	CN Loss	1300000	33	3	30	0.010	5	0	
chr4:116,300,000-117,350,000	q26	CN Loss	1050000	33	3	30	0.010	1	0	
chr4:121,050,000-121,100,000	q27	CN Loss	50000	33	3	30	0.010	0	0	
chr4:123,450,000-123,650,000	q27	CN Loss	200000	33	3	30	0.010	2	0	
chr4:124,300,000-125,100,000	q28.1	CN Gain	800000	33	3	30	0.010	2	0	
chr4:56,150,000-56,450,000	q12	CN Loss	300000	33	3	30	0.010	5	0	
chr4:71,450,000-72,250,000	q13.3	CN Loss	800000	33	3	30	0.010	9	0	
chr9:107,450,000-107,500,000	q31.1	CN Loss	50000	33	3	30	0.010	1	0	
chr9:108,250,000-108,350,000	q31.2	CN Loss	100000	33	3	30	0.010	3	0	
chr9:123,200,000-124,450,000	q33.2	CN Loss	1250000	33	3	30	0.010	17	1	CNTRL
chr8:108,450,000-110,400,000	q23.1	CN Gain	1950000	80	35	45	0.011	9	2	RSPO2, EIF3E
chr8:117,500,000-118,650,000	q23.3 - q24.11	CN Gain	1150000	67	26	41	0.011	9	1	RAD21
chr8:16,750,000-16,850,000	p22	CN Gain	100000	67	26	41	0.011	1	0	
chr8:18,500,000-18,850,000	p22	CN Gain	350000	67	26	41	0.011	1	0	
chr8:97,175,000-99,950,000	q22.1 - q22.2	CN Gain	2775000	67	26	41	0.011	21	0	
chr1:156,200,000-156,550,000	q22 - q23.1	CN Gain	350000	80	39	41	0.012	15	0	
chr8:111,450,000-111,900,000	q23.2	CN Gain	450000	80	39	41	0.012	0	0	
chr1:167,200,000-175,000,000	q24.2 - q25.1	CN Gain	7800000	73	32	41	0.012	101	1	PRRX1
chr1:178,000,000-181,100,000	q25.2 - q25.3	CN Gain	3100000	73	32	41	0.012	33	1	ABL2
chr1:182,100,000-185,100,000	q25.3	CN Gain	3000000	73	32	41	0.012	32	0	
chr1:186,100,000-187,600,000	q31.1	CN Gain	1500000	73	32	41	0.012	11	1	TPR
chr1:196,200,000-206,700,000	q31.3 - q32.1	CN Gain	10500000	73	32	41	0.012	146	5	PTPRC, ELF3, MDM4, ELK4, SLC45A3
chr1:211,400,000-213,300,000	q32.2 - q32.3	CN Gain	1900000	73	32	41	0.012	31	0	
chr1:222,750,000-223,775,000	q41	CN Gain	1025000	73	32	41	0.012	11	0	
chr1:224,850,000-228,750,000	q42.12 - q42.13	CN Gain	3900000	73	32	41	0.012	87	1	H3F3A
chr1:233,050,000-234,450,000	q42.2	CN Gain	1400000	73	32	41	0.012	7	0	
chr11:50,000,000-50,150,000	p11.12	CN Loss	150000	73	32	41	0.012	1	0	
chr8:101,250,000-103,900,000	q22.2 - q22.3	CN Gain	2650000	73	32	41	0.012	25	2	PABPC1, UBR5
chr8:105,800,000-105,850,000	q22.3	CN Gain	50000	73	32	41	0.012	0	0	
chr8:119,000,000-119,050,000	q24.11	CN Gain	50000	73	32	41	0.012	1	1	EXT1
chr8:132,350,000-134,600,000	q24.22	CN Gain	2250000	73	32	41	0.012	15	1	NDRG1
chr13:19,900,000-20,000,000	q12.11	CN Loss	100000	53	16	37	0.014	3	0	
chr22:43,800,000-44,150,000	q13.2	CN Loss	350000	53	16	37	0.014	4	0	
chr22:47,150,000-47,200,000	q13.31	CN Loss	50000	53	16	37	0.014	1	0	
chr22:50,450,000-51,304,566	q13.33	CN Loss	854566	53	16	37	0.014	36	0	
chr3:0-950,000	p26.3	CN Gain	950000	53	16	37	0.014	5	0	
chr6:163,050,000-163,150,000	q26	CN Loss	100000	53	16	37	0.014	2	0	
chr8:27,500,000-28,050,000	p21.1	CN Gain	550000	53	16	37	0.014	10	0	
chr8:31,050,000-31,200,000	p12	CN Gain	150000	53	16	37	0.014	0	0	
chr8:39,250,000-39,350,000	p11.22	CN Gain	100000	53	16	37	0.014	2	0	
chr8:143,550,000-144,100,000	q24.3	CN Gain	550000	60	23	37	0.021	20	0	
chr8:37,600,000-39,200,000	p11.23 - p11.22	CN Gain	1600000	60	23	37	0.021	28	3	NSD3, WHSC1L1, FGFR1
chr11:5,350,000-5,400,000	p15.4	CN Loss	50000	47	13	34	0.024	2	0	
chr11:5,600,000-6,100,000	p15.4	CN Loss	500000	47	13	34	0.024	20	0	
chr14:78,650,000-78,750,000	q24.3	CN Loss	100000	47	13	34	0.024	1	0	
chr14:79,000,000-79,300,000	q24.3 - q31.1	CN Loss	300000	47	13	34	0.024	1	0	
chr16:83,000,000-83,050,000	q23.3	CN Loss	50000	47	13	34	0.024	2	0	
chr20:35,850,000-35,900,000	q11.23	CN Gain	50000	47	13	34	0.024	2	0	
chr22:44,700,000-47,000,000	q13.31	CN Loss	2300000	47	13	34	0.024	42	0	
chr3:148,300,000-148,450,000	q24	CN Loss	150000	47	13	34	0.024	1	0	
chr6:163,650,000-163,950,000	q26	CN Loss	300000	47	13	34	0.024	5	1	QKI
chr8:14,700,000-14,750,000	p22	CN Gain	50000	47	13	34	0.024	2	0	
chr8:141,000,000-141,100,000	q24.3	CN Gain	100000	47	13	34	0.024	1	0	
chr8:22,600,000-24,250,000	p21.3 - p21.2	CN Gain	1650000	47	13	34	0.024	22	0	
chr8:26,000,000-27,400,000	p21.2 - p21.1	CN Gain	1400000	47	13	34	0.024	13	0	
chr8:33,450,000-33,500,000	p12	CN Gain	50000	47	13	34	0.024	1	0	
chr8:50,200,000-51,150,000	q11.21	CN Gain	950000	47	13	34	0.024	2	0	
chr9:103,400,000-103,750,000	q31.1	CN Loss	350000	47	13	34	0.024	0	0	
chr9:104,400,000-105,850,000	q31.1	CN Loss	1450000	47	13	34	0.024	3	0	
chr9:120,450,000-123,100,000	q33.1 - q33.2	CN Loss	2650000	47	13	34	0.024	5	0	
chr9:138,800,000-139,100,000	q34.3	CN Loss	300000	47	13	34	0.024	5	0	
chrX:127,800,000-128,300,000	q25	CN Loss	500000	47	13	34	0.024	0	0	
chrX:137,950,000-139,800,000	q26.3 - q27.1	CN Loss	1850000	47	13	34	0.024	11	0	
chrX:6,550,000-7,350,000	p22.31	CN Loss	800000	47	13	34	0.024	3	0	
chr1:181,250,000-181,700,000	q25.3	CN Gain	450000	67	29	38	0.025	1	0	
chr1:213,350,000-213,450,000	q32.3	CN Gain	100000	67	29	38	0.025	1	0	
chr1:26,300,000-26,600,000	p36.11	CN Loss	300000	67	29	38	0.025	12	0	
chr1:26,800,000-26,900,000	p36.11	CN Loss	100000	67	29	38	0.025	3	0	
chr1:9,550,000-10,550,000	p36.22	CN Loss	1000000	67	29	38	0.025	25	0	
chr13:19,275,000-19,750,000	q11 - q12.11	CN Loss	475000	67	29	38	0.025	6	0	
chr8:115,400,000-117,500,000	q23.3	CN Gain	2100000	67	29	38	0.025	2	0	
chr8:118,650,000-118,750,000	q24.11	CN Gain	100000	67	29	38	0.025	0	0	
chr8:135,000,000-135,400,000	q24.22	CN Gain	400000	67	29	38	0.025	0	0	
chr8:144,400,000-145,000,000	q24.3	CN Gain	600000	67	29	38	0.025	32	0	
chr8:16,850,000-17,350,000	p22	CN Gain	500000	67	29	38	0.025	6	0	
chr8:18,100,000-18,500,000	p22	CN Gain	400000	67	29	38	0.025	2	0	
chr8:112,500,000-112,550,000	q23.3	CN Gain	50000	80	42	38	0.026	0	0	
chr1:156,800,000-156,950,000	q23.1	CN Gain	150000	73	35	38	0.027	6	1	NTRK1
chr1:223,775,000-224,850,000	q41 - q42.12	CN Gain	1075000	73	35	38	0.027	15	0	
chr1:23,400,000-23,650,000	p36.12	CN Loss	250000	73	35	38	0.027	5	0	
chr1:234,450,000-236,150,000	q42.2 - q42.3	CN Gain	1700000	73	35	38	0.027	23	0	
chr11:49,950,000-50,000,000	p11.12	CN Loss	50000	73	35	38	0.027	1	0	
chr8:141,750,000-142,400,000	q24.3	CN Gain	650000	73	35	38	0.027	6	0	

chr10:22,200,000-22,300,000	p12.31	CN Gain	100000	33	6	27	0.029	1	0	
chr10:24,250,000-25,200,000	p12.2 - p12.1	CN Gain	950000	33	6	27	0.029	4	0	
chr10:3,000,000-3,950,000	p15.2 - p15.1	CN Gain	950000	33	6	27	0.029	10	1	KLF6
chr10:59,800,000-59,850,000	q21.1	CN Gain	50000	33	6	27	0.029	0	0	
chr10:60,550,000-61,550,000	q21.1 - q21.2	CN Gain	1000000	33	6	27	0.029	8	1	CCDC6
chr10:64,850,000-64,900,000	q21.3	CN Gain	50000	33	6	27	0.029	1	0	
chr10:73,350,000-73,550,000	q22.1	CN Gain	200000	33	6	27	0.029	3	0	
chr10:77,200,000-77,450,000	q22.2	CN Gain	250000	33	6	27	0.029	2	0	
chr10:94,550,000-94,650,000	q23.33	CN Gain	100000	33	6	27	0.029	1	0	
chr11:20,600,000-20,900,000	p15.1	CN Loss	300000	33	6	27	0.029	2	0	
chr11:7,550,000-7,950,000	p15.4	CN Loss	400000	33	6	27	0.029	8	0	
chr12:103,300,000-103,450,000	q23.2	CN Loss	150000	33	6	27	0.029	2	0	
chr12:104,000,000-104,050,000	q23.3	CN Loss	50000	33	6	27	0.029	1	0	
chr13:20,200,000-20,250,000	q12.11	CN Loss	50000	33	6	27	0.029	2	0	
chr13:44,400,000-45,150,000	q14.11	CN Loss	750000	33	6	27	0.029	11	0	
chr13:53,450,000-54,000,000	q14.3	CN Loss	550000	33	6	27	0.029	2	0	
chr13:68,450,000-69,300,000	q21.32 - q21.33	CN Loss	850000	33	6	27	0.029	0	0	
chr13:72,800,000-74,700,000	q21.33 - q22.1	CN Gain	1900000	33	6	27	0.029	7	0	
chr13:78,500,000-78,950,000	q22.3	CN Loss	450000	33	6	27	0.029	4	0	
chr13:86,850,000-87,950,000	q31.1 - q31.2	CN Gain	1100000	33	6	27	0.029	1	0	
chr14:23,800,000-24,450,000	q11.2	CN Loss	650000	33	6	27	0.029	19	0	
chr14:25,000,000-25,100,000	q12	CN Loss	100000	33	6	27	0.029	2	0	
chr14:26,050,000-26,600,000	q12	CN Loss	550000	33	6	27	0.029	0	0	
chr14:28,900,000-29,900,000	q12	CN Loss	1000000	33	6	27	0.029	4	0	
chr14:36,450,000-37,450,000	q13.2 - q13.3	CN Loss	1000000	33	6	27	0.029	10	1	NKX2-1
chr14:53,750,000-54,350,000	q22.1 - q22.2	CN Loss	600000	33	6	27	0.029	0	0	
chr14:55,900,000-58,550,000	q22.3 - q23.1	CN Loss	2650000	33	6	27	0.029	16	1	KTN1
chr14:59,500,000-60,000,000	q23.1	CN Loss	500000	33	6	27	0.029	5	0	
chr14:65,400,000-66,200,000	q23.3	CN Loss	800000	33	6	27	0.029	13	1	MAX
chr14:66,850,000-66,900,000	q23.3	CN Loss	50000	33	6	27	0.029	0	0	
chr14:69,850,000-70,200,000	q24.1 - q24.2	CN Loss	350000	33	6	27	0.029	5	0	
chr14:70,600,000-71,950,000	q24.2	CN Loss	1350000	33	6	27	0.029	17	0	
chr14:79,700,000-80,150,000	q31.1	CN Loss	450000	33	6	27	0.029	1	0	
chr15:60,000,000-61,100,000	q22.2	CN Loss	1100000	33	6	27	0.029	6	0	
chr16:73,250,000-73,500,000	q22.3	CN Loss	250000	33	6	27	0.029	1	0	
chr16:74,200,000-74,250,000	q23.1	CN Loss	50000	33	6	27	0.029	1	0	
chr16:80,425,000-80,750,000	q23.2	CN Loss	325000	33	6	27	0.029	4	0	
chr16:81,800,000-81,925,000	q23.3	CN Loss	125000	33	6	27	0.029	1	0	
chr17:41,350,000-41,650,000	q21.31	CN Gain	300000	33	6	27	0.029	9	1	ETV4
chr17:47,850,000-47,950,000	q21.33	CN Gain	100000	33	6	27	0.029	3	1	KAT7
chr17:57,050,000-57,150,000	q22	CN Gain	100000	33	6	27	0.029	2	0	
chr17:74,800,000-75,450,000	q25.2 - q25.3	CN Gain	650000	33	6	27	0.029	11	1	40057
chr19:55,500,000-56,200,000	q13.42	CN Loss	700000	33	6	27	0.029	46	0	
chr2:164,100,000-164,950,000	q24.3	CN Gain	850000	33	6	27	0.029	1	0	
chr3:101,600,000-101,800,000	q12.3	CN Gain	200000	33	6	27	0.029	1	0	
chr3:105,250,000-105,400,000	q13.11	CN Gain	150000	33	6	27	0.029	2	1	CBLB
chr3:111,250,000-112,000,000	q13.13 - q13.2	CN Gain	750000	33	6	27	0.029	13	0	
chr3:123,950,000-124,400,000	q21.2	CN Gain	450000	33	6	27	0.029	2	0	
chr3:152,200,000-152,500,000	q25.2	CN Gain	300000	33	6	27	0.029	0	0	
chr3:170,100,000-171,550,000	q26.2 - q26.31	CN Gain	1450000	33	6	27	0.029	11	0	
chr3:179,000,000-179,100,000	q26.33	CN Gain	100000	33	6	27	0.029	2	0	
chr3:23,300,000-24,950,000	p24.3 - p24.2	CN Gain	1650000	33	6	27	0.029	11	0	
chr3:31,250,000-32,200,000	p23 - p22.3	CN Gain	950000	33	6	27	0.029	5	0	
chr3:98,500,000-99,350,000	q12.1	CN Gain	850000	33	6	27	0.029	2	0	
chr4:101,600,000-101,850,000	q24	CN Loss	250000	33	6	27	0.029	0	0	
chr4:109,350,000-110,650,000	q25	CN Loss	1300000	33	6	27	0.029	13	0	
chr4:115,050,000-116,300,000	q26	CN Loss	1250000	33	6	27	0.029	3	0	
chr4:121,100,000-122,250,000	q27	CN Loss	1150000	33	6	27	0.029	4	0	
chr4:130,000,000-130,350,000	q28.2	CN Loss	350000	33	6	27	0.029	2	0	
chr4:130,900,000-131,200,000	q28.2 - q28.3	CN Loss	300000	33	6	27	0.029	0	0	
chr4:132,450,000-132,550,000	q28.3	CN Loss	100000	33	6	27	0.029	0	0	
chr4:14,800,000-15,450,000	p15.33 - p15.32	CN Loss	650000	33	6	27	0.029	5	0	
chr4:141,500,000-142,100,000	q31.21	CN Gain	600000	33	6	27	0.029	3	0	
chr4:147,100,000-147,250,000	q31.22	CN Loss	150000	33	6	27	0.029	3	0	
chr4:147,900,000-149,500,000	q31.22 - q31.23	CN Loss	1600000	33	6	27	0.029	7	0	
chr4:151,900,000-153,150,000	q31.3	CN Loss	1250000	33	6	27	0.029	9	0	
chr4:165,650,000-166,650,000	q32.3	CN Loss	1000000	33	6	27	0.029	12	0	
chr4:169,050,000-169,800,000	q32.3	CN Loss	750000	33	6	27	0.029	4	0	
chr4:17,200,000-19,400,000	p15.32 - p15.31	CN Loss	2200000	33	6	27	0.029	9	0	
chr4:2,500,000-4,600,000	p16.3 - p16.2	CN Loss	2100000	33	6	27	0.029	28	0	
chr4:21,000,000-21,800,000	p15.31 - p15.2	CN Loss	800000	33	6	27	0.029	3	0	
chr4:24,050,000-25,950,000	p15.2	CN Loss	1900000	33	6	27	0.029	15	1	SLC34A2
chr4:27,000,000-27,650,000	p15.2	CN Loss	650000	33	6	27	0.029	2	0	
chr4:34,100,000-36,400,000	p15.1 - p14	CN Loss	2300000	33	6	27	0.029	4	0	
chr4:41,550,000-42,100,000	p13	CN Loss	550000	33	6	27	0.029	6	1	PHOX2B
chr4:48,100,000-49,225,000	p12 - p11	CN Loss	1125000	33	6	27	0.029	10	1	TEC
chr4:53,900,000-54,750,000	q12	CN Loss	850000	33	6	27	0.029	6	1	FIP1L1
chr4:58,050,000-58,450,000	q12	CN Loss	400000	33	6	27	0.029	2	0	
chr4:6,650,000-8,550,000	p16.1	CN Loss	1900000	33	6	27	0.029	27	0	
chr4:60,700,000-66,350,000	q13.1	CN Loss	5650000	33	6	27	0.029	7	0	
chr4:67,300,000-68,950,000	q13.2	CN Loss	1650000	33	6	27	0.029	12	0	
chr4:69,800,000-71,450,000	q13.2 - q13.3	CN Loss	1650000	33	6	27	0.029	28	0	
chr4:72,250,000-75,950,000	q13.3	CN Loss	3700000	33	6	27	0.029	29	0	
chr4:76,400,000-76,550,000	q21.1	CN Loss	150000	33	6	27	0.029	4	0	
chr4:77,700,000-77,800,000	q21.1	CN Loss	100000	33	6	27	0.029	1	0	
chr4:79,750,000-79,850,000	q21.21	CN Loss	100000	33	6	27	0.029	2	0	
chr4:83,200,000-85,600,000	q21.22 - q21.23	CN Loss	2400000	33	6	27	0.029	25	0	
chr4:96,400,000-97,000,000	q22.3	CN Loss	600000	33	6	27	0.029	2	0	
chr4:97,100,000-98,450,000	q22.3	CN Loss	1350000	33	6	27	0.029	2	0	
chr4:99,000,000-100,950,000	q23	CN Loss	1950000	33	6	27	0.029	25	1	RAP1GDS1
chr5:142,450,000-142,500,000	q31.3	CN Gain	50000	33	6	27	0.029	1	1	ARHGAP26
chr5:16,450,000-19,900,000	p15.1 - p14.3	CN Gain	3450000	33	6	27	0.029	14	0	
chr5:30,550,000-31,300,000	p13.3	CN Gain	750000	33	6	27	0.029	1	0	
chr5:38,450,000-39,300,000	p13.1	CN Gain	850000	33	6	27	0.029	10	1	LIFR
chr5:53,000,000-53,100,000	q11.2	CN Gain	100000	33	6	27	0.029	1	0	
chr5:53,350,000-56,550,000	q11.2	CN Gain	3200000	33	6	27	0.029	34	2	IL6ST, MAP3K1
chr5:66,200,000-67,400,000	q12.3 - q13.1	CN Gain	1200000	33	6	27	0.029	5	0	
chr6:25,950,000-27,050,000	p22.2 - p22.1	CN Gain	1100000	33	6	27	0.029	51	1	HIST1H3B
chr8:20,300,000-20,850,000	p21.3	CN Gain	550000	33	6	27	0.029	3	0	
chr8:35,000,000-36,650,000	p12 - p11.23	CN Gain	1650000	33	6	27	0.029	3	0	
chr9:108,350,000-108,400,000	q31.2	CN Loss	50000	33	6	27	0.029	1	0	
chr9:109,350,000-109,950,000	q31.2	CN Loss	600000	33	6	27	0.029	4	0	
chr9:114,950,000-115,450,000	q32	CN Loss	500000	33	6	27	0.029	5	0	
chr9:73,250,000-73,550,000	q21.12	CN Loss	300000	33	6	27	0.029	2	0	
chrX:2,000,000-2,075,000	p22.33	CN Loss	75000	33	6	27	0.029	0	0	
chrX:29,200,000-31,050,000	p21.3 - p21.2	CN Loss	1850000	33	6	27	0.029	11	0	
chrX:32,050,000-32,550,000	p21.1	CN Loss	500000	33	6	27	0.029	1	0	
chrX:4,000,000-4,850,000	p22.33 - p22.32	CN Loss	850000	33	6	27	0.029	1	0	
chrX:5,400,000-5,800,000	p22.32	CN Loss	400000	33	6	27	0.029	0	0	

chr1:145,100,000-147,825,000	q21.1 - q21.2	CN Gain	2725000	80	45	35	0.031	59	1	BCL9
chr1:156,100,000-156,200,000	q22	CN Gain	100000	80	45	35	0.031	5	1	LMNA
chr8:112,550,000-113,150,000	q23.3	CN Gain	600000	80	45	35	0.031	0	0	
chr11:5,400,000-5,600,000	p15.4	CN Loss	200000	47	16	31	0.038	11	0	
chr14:78,750,000-79,000,000	q24.3	CN Loss	250000	47	16	31	0.038	1	0	
chr16:83,050,000-83,700,000	q23.3	CN Loss	650000	47	16	31	0.038	2	0	
chr16:86,200,000-86,500,000	q24.1	CN Loss	300000	47	16	31	0.038	4	0	
chr20:11,250,000-11,350,000	p12.2	CN Gain	100000	47	16	31	0.038	1	0	
chr20:32,150,000-35,850,000	q11.22 - q11.23	CN Gain	3700000	47	16	31	0.038	74	0	
chr20:9,700,000-10,250,000	p12.2	CN Gain	550000	47	16	31	0.038	5	0	
chr22:25,600,000-26,500,000	q11.23 - q12.1	CN Loss	900000	47	16	31	0.038	8	0	
chr22:27,125,000-27,600,000	q12.1	CN Loss	475000	47	16	31	0.038	5	0	
chr22:43,600,000-43,800,000	q13.2	CN Loss	200000	47	16	31	0.038	4	0	
chr22:44,150,000-44,700,000	q13.2 - q13.31	CN Loss	550000	47	16	31	0.038	8	0	
chr22:47,000,000-47,150,000	q13.31	CN Loss	150000	47	16	31	0.038	2	0	
chr3:148,450,000-148,700,000	q24	CN Gain	250000	47	16	31	0.038	3	0	
chr3:191,050,000-191,100,000	q28	CN Gain	50000	47	16	31	0.038	1	0	
chr3:196,350,000-198,022,430	q29	CN Gain	1672430	47	16	31	0.038	29	0	
chr5:24,400,000-25,900,000	p14.2 - p14.1	CN Gain	1500000	47	16	31	0.038	4	1	CDH10
chr6:160,450,000-163,050,000	q25.3 - q26	CN Loss	2600000	47	16	31	0.038	13	0	
chr6:163,150,000-163,650,000	q26	CN Loss	500000	47	16	31	0.038	3	0	
chr6:163,950,000-164,500,000	q26 - q27	CN Loss	550000	47	16	31	0.038	2	1	QKI
chr8:140,900,000-141,000,000	q24.3	CN Gain	100000	47	16	31	0.038	1	0	
chr8:21,850,000-22,600,000	p21.3	CN Gain	750000	47	16	31	0.038	27	0	
chr8:31,200,000-31,450,000	p12	CN Gain	250000	47	16	31	0.038	0	0	
chr8:32,750,000-33,450,000	p12	CN Gain	700000	47	16	31	0.038	6	0	
chr8:37,150,000-37,400,000	p11.23	CN Gain	250000	47	16	31	0.038	3	0	
chr8:51,150,000-51,900,000	q11.21	CN Gain	750000	47	16	31	0.038	1	0	
chr8:52,550,000-53,700,000	q11.22 - q11.23	CN Gain	1150000	47	16	31	0.038	6	0	
chr9:0-600,000	p24.3	CN Loss	600000	47	16	31	0.038	15	0	
chr9:119,450,000-120,450,000	q33.1	CN Loss	1000000	47	16	31	0.038	4	0	
chr9:137,550,000-138,800,000	q34.3	CN Loss	1250000	47	16	31	0.038	28	0	
chrX:117,200,000-117,400,000	q24	CN Loss	200000	47	16	31	0.038	1	0	
chrX:150,100,000-151,800,000	q28	CN Loss	1700000	47	16	31	0.038	22	0	
chrX:64,400,000-67,250,000	q11.2 - q12	CN Loss	2850000	47	16	31	0.038	9	2	MSN, AR
chr12:120,700,000-121,850,000	q24.31	CN Gain	1150000	53	19	34	0.038	31	1	HNF1A
chr13:19,850,000-19,900,000	q12.11	CN Loss	50000	53	19	34	0.038	1	0	
chr22:35,500,000-35,550,000	q12.3	CN Loss	50000	53	19	34	0.038	1	0	
chr22:47,200,000-47,250,000	q13.31	CN Loss	50000	53	19	34	0.038	1	0	
chr22:50,300,000-50,450,000	q13.33	CN Loss	150000	53	19	34	0.038	5	0	
chr3:148,700,000-149,650,000	q24 - q25.1	CN Gain	950000	53	19	34	0.038	14	1	WWTR1
chr8:142,450,000-143,050,000	q24.3	CN Gain	600000	53	19	34	0.038	2	0	
chr8:37,400,000-37,450,000	p11.23	CN Gain	50000	53	19	34	0.038	0	0	
chr8:51,950,000-52,550,000	q11.21 - q11.22	CN Gain	600000	53	19	34	0.038	1	0	
chr8:66,550,000-68,350,000	q13.1 - q13.2	CN Gain	1800000	53	19	34	0.038	26	0	
chr9:140,675,000-140,750,000	q34.3	CN Loss	75000	53	19	34	0.038	3	0	
chr9:7,100,000-8,550,000	p24.1	CN Loss	1450000	53	19	34	0.038	3	1	PTPRD
chr3:134,400,000-134,450,000	q22.2	CN Loss	50000	0	26	26	0.040	0	0	
chr10:75,600,000-77,200,000	q22.2	CN Gain	1600000	40	10	30	0.042	20	1	KAT6B
chr11:4,850,000-5,300,000	p15.4	CN Loss	450000	40	10	30	0.042	24	0	
chr12:103,500,000-103,950,000	q23.2 - q23.3	CN Loss	450000	40	10	30	0.042	4	0	
chr13:54,850,000-55,400,000	q14.3 - q21.1	CN Loss	550000	40	10	30	0.042	1	0	
chr14:79,300,000-79,400,000	q31.1	CN Loss	100000	40	10	30	0.042	1	0	
chr16:82,200,000-82,400,000	q23.3	CN Loss	200000	40	10	30	0.042	1	0	
chr17:72,950,000-73,950,000	q25.1	CN Gain	1000000	40	10	30	0.042	41	1	H3F3B
chr19:51,650,000-54,300,000	q13.41 - q13.42	CN Loss	2650000	40	10	30	0.042	147	2	PPP2R1A, ZNF331
chr19:56,300,000-57,050,000	q13.43	CN Loss	750000	40	10	30	0.042	20	0	
chr2:101,150,000-101,300,000	q11.2	CN Gain	150000	40	10	30	0.042	2	0	
chr20:36,000,000-36,100,000	q11.23	CN Gain	100000	40	10	30	0.042	1	1	SRC
chr22:32,350,000-32,500,000	q12.3	CN Loss	150000	40	10	30	0.042	3	0	
chr22:35,650,000-36,950,000	q12.3	CN Loss	1300000	40	10	30	0.042	20	1	MYH9
chr22:37,850,000-39,950,000	q13.1	CN Loss	2100000	40	10	30	0.042	73	2	APOBEC3B, PDGFB
chr22:43,350,000-43,400,000	q13.2	CN Loss	50000	40	10	30	0.042	1	0	
chr3:124,450,000-125,450,000	q21.2	CN Gain	1000000	40	10	30	0.042	10	0	
chr3:175,150,000-176,300,000	q26.31 - q26.32	CN Gain	1150000	40	10	30	0.042	3	0	
chr3:178,150,000-178,350,000	q26.32	CN Gain	200000	40	10	30	0.042	3	0	
chr4:10,150,000-11,150,000	p16.1	CN Loss	1000000	40	10	30	0.042	2	0	
chr4:11,750,000-14,450,000	p15.33	CN Loss	2700000	40	10	30	0.042	11	0	
chr4:147,350,000-147,900,000	q31.22	CN Loss	550000	40	10	30	0.042	3	0	
chr4:164,200,000-165,550,000	q32.2 - q32.3	CN Loss	1350000	40	10	30	0.042	6	0	
chr4:167,075,000-168,700,000	q32.3	CN Loss	1625000	40	10	30	0.042	1	0	
chr4:170,200,000-171,000,000	q33	CN Loss	800000	40	10	30	0.042	7	0	
chr4:182,300,000-186,050,000	q34.3 - q35.1	CN Loss	3750000	40	10	30	0.042	34	1	CASP3
chr4:36,550,000-37,400,000	p14	CN Loss	850000	40	10	30	0.042	4	0	
chr4:37,500,000-37,550,000	p14	CN Loss	50000	40	10	30	0.042	1	0	
chr4:42,100,000-42,800,000	p13	CN Loss	700000	40	10	30	0.042	4	0	
chr4:43,500,000-43,800,000	p13	CN Loss	300000	40	10	30	0.042	0	0	
chr4:52,725,000-53,400,000	q12	CN Loss	675000	40	10	30	0.042	5	0	
chr4:66,450,000-67,150,000	q13.1 - q13.2	CN Loss	700000	40	10	30	0.042	3	0	
chr5:140,850,000-142,450,000	q31.3	CN Gain	1600000	40	10	30	0.042	42	1	ARHGAP26
chr5:23,350,000-23,400,000	p14.2	CN Gain	50000	40	10	30	0.042	0	0	
chr5:26,500,000-27,500,000	p14.1	CN Gain	1000000	40	10	30	0.042	2	0	
chr5:67,450,000-67,550,000	q13.1	CN Gain	100000	40	10	30	0.042	2	1	PIK3R1
chr6:103,400,000-104,300,000	q16.3	CN Loss	900000	40	10	30	0.042	0	0	
chr6:93,750,000-95,750,000	q16.1	CN Loss	2000000	40	10	30	0.042	2	1	EPHA7
chr8:24,350,000-24,400,000	p21.2	CN Gain	50000	40	10	30	0.042	3	0	
chr8:34,000,000-34,800,000	p12	CN Gain	800000	40	10	30	0.042	1	0	
chr8:36,700,000-36,800,000	p11.23	CN Gain	100000	40	10	30	0.042	1	0	
chr9:106,700,000-106,800,000	q31.1	CN Loss	100000	40	10	30	0.042	1	0	
chr9:124,650,000-128,600,000	q33.2 - q33.3	CN Loss	3950000	40	10	30	0.042	61	1	PPP6C
chr9:139,250,000-140,550,000	q34.3	CN Loss	1300000	40	10	30	0.042	89	1	NOTCH1
chr9:73,600,000-74,200,000	q21.12 - q21.13	CN Loss	600000	40	10	30	0.042	1	0	
chrX:0-1,350,000	p22.33	CN Loss	1350000	40	10	30	0.042	6	1	CRLF2
chrX:139,950,000-140,700,000	q27.1 - q27.2	CN Loss	750000	40	10	30	0.042	14	0	
chrX:14,950,000-18,900,000	p22.2 - p22.13	CN Loss	3950000	40	10	30	0.042	38	1	ZRSR2
chrX:24,550,000-25,700,000	p22.11 - p21.3	CN Loss	1150000	40	10	30	0.042	6	0	
chrX:26,500,000-29,200,000	p21.3	CN Loss	2700000	40	10	30	0.042	7	0	
chrX:6,000,000-6,450,000	p22.31	CN Loss	450000	40	10	30	0.042	3	0	
chrX:7,650,000-8,250,000	p22.31	CN Loss	600000	40	10	30	0.042	4	0	
chrX:9,600,000-10,950,000	p22.2	CN Loss	1350000	40	10	30	0.042	7	0	
chr19:43,100,000-43,550,000	q13.2 - q13.31	CN Loss	450000	60	26	34	0.048	9	0	
chr22:34,350,000-35,400,000	q12.3	CN Loss	1050000	60	26	34	0.048	1	0	
chr22:47,250,000-47,300,000	q13.31	CN Loss	50000	60	26	34	0.048	1	0	
chr8:136,075,000-137,750,000	q24.22 - q24.23	CN Gain	1675000	60	26	34	0.048	3	0	
chr8:137,900,000-138,400,000	q24.23	CN Gain	500000	60	26	34	0.048	1	0	
chr8:138,500,000-138,750,000	q24.23	CN Gain	250000	60	26	34	0.048	0	0	
chr8:144,100,000-144,250,000	q24.3	CN Gain	150000	60	26	34	0.048	4	0	
chr8:47,125,000-48,700,000	q11.1 - q11.21	CN Gain	1575000	60	26	34	0.048	6	0	
chr8:94,150,000-97,175,000	q22.1	CN Gain	3025000	60	26	34	0.048	29	1	CDH17

chr12:120,250,000-120,700,000	q24.23 - q24.31	CN Gain	450000	53	23	31	0.050	8	0	
chr12:121,850,000-122,575,000	q24.31	CN Gain	725000	53	23	31	0.050	16	2	SETD1B, BCL7A
chr13:19,750,000-19,850,000	q12.11	CN Loss	100000	53	23	31	0.050	3	0	
chr22:34,100,000-34,350,000	q12.3	CN Loss	250000	53	23	31	0.050	3	0	
chr8:139,050,000-139,400,000	q24.23	CN Gain	350000	53	23	31	0.050	2	1	FAM135B
chr8:39,200,000-39,250,000	p11.22	CN Gain	50000	53	23	31	0.050	1	0	
chr8:48,975,000-49,150,000	q11.21	CN Gain	175000	53	23	31	0.050	1	0	
chr9:140,750,000-141,213,431	q34.3	CN Loss	463431	53	23	31	0.050	6	0	

Supplementary table 3. Enrichment of genes belonging to each pathway comparing PR-low to PR-high LGSOCs.

To determine whether known cancer genes affected within regions of significant CN differences comparing PR-low compared to PR-high LGSOCs (As shown in supplemental table 3: n=113/729 cancer genes listed in Cosmic) were enriched within common signalling pathways, pathway enrichment analysis was performed using Metascape pathway analysis. From our 113 affected gene list, the number of genes belonging to each cancer signalling pathway was determined. The top 5 cancer signalling pathways and associated Log10(P) value are shown here and visualised in figure 1c.

**Cancer signalling pathway**	**Number of protein encoding genes linked to pathway**	**Proteins**	**Log10(P)**
Diseases of signal transduction by growth factor receptors and second messengers	23/433	FGFR1,FLT3,KDR,KIT,LMNA,MSN,MYC,MYH9,NOTCH1,PDGFB,PDGFRA,PIK3R1,PPP2R1A,RAF1,SRC,TPR,ZMYM2,QKI,CNTRL,RNF43,FBXW7,RNF213,FIP1L1	-18.92890833
Peptidyl-tyrosine kinase phosphorylation and modification	18/376	ABL2,CBLB,EPHA7,FGFR1,FLT3,IL2,IL6ST,KDR,KIT,NTRK1,PDGFB,PDGFRA,PPP2R1A,PTPRC,MAP2K4,SRC,TEC,FBXW7,CDH10,CDH17,LATS2,LEF1,MYC,PIK3R1,RAF1,GATA3,ETV4,BRCA2,MDM4,LIFR,AR,PTPRD,FIP1L1,PPARG,SLC45A3,MYH9,MSN	-14.03020608
Transcriptional dysregulation of Hippo-Merlin signaling	12/180	ELK4,ETV4,FLT3,H3-3A,H3-3B,MAX,MYC,NTRK1,PPARG,H3C2,ASPSCR1,SLC45A3,BRCA2,SRC,WRN,RECQL4,DEK,LEF1,BCL9,LMNA,RAD21,PPP2R1A,TPR,GATA3,ELF3,NOTCH1,AR,MYH9,DAXX,PPP6C,MDM4,MAP2K4,SETD1B	-11.13566843
PI3K signaling	10/142	FGFR1,FLT3,GATA3,KDR,KIT,NTRK1,PDGFB,PDGFRA,PIK3R1,SRC	-9.588748829

MAPK signaling	12/255	CASP3,DAXX,ELK4,FGFR1,MAX,MAP3K1,MYC,NTRK1,PDGFB,PDGFRA,RAF1,MAP2K4,LMNA,PIK3R1,PRF1,CBLB,EPHA7,FBXW7	-9.378039643
