# Supplementary material for: Molecular characterization of low-grade serous ovarian carcinoma identifies genomic aberrations according to hormone receptor expression
Source: NPJ Precis Oncol. 2022 Jun 29;6:47. doi: 10.1038/s41698-022-00288-2 (PMC9242985; doi:10.1038/s41698-022-00288-2)
Supplement: Supplementary file 2 — REPORTING SUMMARY [file 41698_2022_288_MOESM2_ESM.pdf]

nature portfolio

Corresponding author(s): Dane Cheasley
Last updated by author(s): Apr 12, 2022

Reporting Summary

Nature Portfolio wishes to improve the reproducibility of the work that we publish. This form provides structure for consistency and transparency in reporting. For further information on Nature Portfolio policies, see our Editorial Policies and the Editorial Policy Checklist.

Statistics

For all statistical analyses, confirm that the following items are present in the figure legend, table legend, main text, or Methods section.

n/a	Confirmed	
☐	☒	The exact sample size (n) for each experimental group/condition, given as a discrete number and unit of measurement
☐	☒	A statement on whether measurements were taken from distinct samples or whether the same sample was measured repeatedly
☐	☒	The statistical test(s) used AND whether they are one- or two-sided<br>*Only common tests should be described solely by name; describe more complex techniques in the Methods section.*
☐	☒	A description of all covariates tested
☐	☒	A description of any assumptions or corrections, such as tests of normality and adjustment for multiple comparisons
☐	☒	A full description of the statistical parameters including central tendency (e.g. means) or other basic estimates (e.g. regression coefficient) AND variation (e.g. standard deviation) or associated estimates of uncertainty (e.g. confidence intervals)
☐	☒	For null hypothesis testing, the test statistic (e.g. F, t, r) with confidence intervals, effect sizes, degrees of freedom and P value noted<br>*Give P values as exact values whenever suitable.*
☒	☐	For Bayesian analysis, information on the choice of priors and Markov chain Monte Carlo settings
☒	☐	For hierarchical and complex designs, identification of the appropriate level for tests and full reporting of outcomes
☒	☐	Estimates of effect sizes (e.g. Cohen's d, Pearson's r), indicating how they were calculated

Our web collection on statistics for biologists contains articles on many of the points above.

Software and code

Policy information about availability of computer code

Data collection	No custom codes were used for data collection
Data analysis	No custom codes were used for data analysis

For manuscripts utilizing custom algorithms or software that are central to the research but not yet described in published literature, software must be made available to editors and reviewers. We strongly encourage code deposition in a community repository (e.g. GitHub). See the Nature Portfolio guidelines for submitting code & software for further information.

Data

Policy information about availability of data

All manuscripts must include a data availability statement. This statement should provide the following information, where applicable:
- Accession codes, unique identifiers, or web links for publicly available datasets
- A description of any restrictions on data availability
- For clinical datasets or third party data, please ensure that the statement adheres to our policy

The datasets analyzed during the current study is available from the corresponding author. Requests for access is subject to review by the COEUR Study Committee.

Field-specific reporting

Please select the one below that is the best fit for your research. If you are not sure, read the appropriate sections before making your selection.

☒ Life sciences ☐ Behavioural & social sciences ☐ Ecological, evolutionary & environmental sciences

For a reference copy of the document with all sections, see nature.com/documents/nr-reporting-summary-flat.pdf

Life sciences study design

All studies must disclose on these points even when the disclosure is negative.

Sample size	A total of 55 primary stage III & IV low-grade serous ovarian cancer samples were analysed in the current study. This represents the total number of cases acquired through the Canadian Ovarian Experimental Unified Resource (COEUR) repository. Given the rarity of the disease and the difficulty in acquiring tissue we analyzed all available samples.
Data exclusions	Given we were interested in poor outcome tumours, stage I and II low-grade serous ovarian cancer samples collected by COEUR were excluded from the study. This is stated in the mansucript
Replication	The raw sequencing data, clinical data and hormone receptor immunohistochemistry can be requested for access by the COEUR Study Committee if researchers want to replicate copy-number data, mutation filtering or investigate individual cases.
Randomization	Following Allred immunohistochemistry scoring for estrogen (ER) and progesterone receptor (PR) expression, which was scored independently by two pathologists and averaged (stated in the paper), low-grade serous cases that were ER-low expression were compared to ER-high, and those cases that were PR-low were compared to PR-high.
Blinding	Somatic mutation, copy-number and fraction of the genome altered scoring was performed blinded to hormone receptor status

Reporting for specific materials, systems and methods

We require information from authors about some types of materials, experimental systems and methods used in many studies. Here, indicate whether each material, system or method listed is relevant to your study. If you are not sure if a list item applies to your research, read the appropriate section before selecting a response.

Materials & experimental systems

n/a	Involved in the study	
☐	☒	Antibodies
☒	☐	Eukaryotic cell lines
☒	☐	Palaeontology and archaeology
☐	☐	Animals and other organisms
☐	☒	Human research participants
☒	☐	Clinical data
☒	☐	Dual use research of concern

Methods

n/a	Involved in the study	
☒	☐	ChIP-seq
☒	☐	Flow cytometry
☒	☐	MRI-based neuroimaging

Antibodies

Antibodies used	The antibodies used in this study included anti-ER antibody SP1 (Cat. No. RM-9101, LabVision, Fremont, CA; 1:200 dilution), anti-PR antibody clone E12 (Cat. No. 790-2223, Ventana Medical Systems Inc., Tucson, AZ; 1:200 dilution), anti-p16 (E6H4 clone, CINtec, mtm laboratories; 1:24 dilution) and anti-TP53 (clone DO-7, Leica Biosystems, Buffalo Grove, IL; 1:5000 dilution).
Validation	All antibodies selected for this study are widely used in clinical diagnosis of breast and ovarian cancer. Importantly, all antibodies are the recommended antibodies by the American Society of Clinical Oncology/College of American Pathologists guidelines and Canadian Association of Pathologists National Standards Committee/Immunohistochemistry. Furthermore, these antibodies were selected and validated by the large Ovarian Tumor Tissue Analysis consortium study (OTTA) looking into associations between genetic abnormalities and altered hormone receptor status to subtype specific ovarian cancer survival.

Animals and other organisms

Policy information about studies involving animals; ARRIVE guidelines recommended for reporting animal research

Laboratory animals	N/A
Wild animals	N/A

Field-collected samples	N/A
Ethics oversight	N/A

Note that full information on the approval of the study protocol must also be provided in the manuscript.

Human research participants

Policy information about studies involving human research participants

Population characteristics	Women diagnosed in Canada with low-grade serous ovarian carcinoma. Median age at diagnosis of 56 years (range 25-77 years)
Recruitment	The COEUR repository has attempted to remove potential centre bias, and our 55 samples have been collected from 10 treatment hospitals around Canada.
Ethics oversight	The hospital biobanks received ethics approval from their local review boards to collect and share samples and clinical data. The collection of the COEUR repository samples and data received local ethics approval by the Comité d'éthique de la recherche du CHUM. Tumor sequencing was approved by the Peter MacCallum Cancer Centre Human Ethics Committee. ER and PR immunohistochemistry and scoring was approved by the institutional human ethics review board at BC Cancer and the University of British Columbia. This information is providing in the manscript, including approval numbers.

Note that full information on the approval of the study protocol must also be provided in the manuscript.
